# Plant ontogeny determines strength and associated plant fitness consequences of plant‐mediated interactions between herbivores and flower visitors

**DOI:** 10.1111/1365-2745.13370

**Published:** 2020-04-04

**Authors:** Quint Rusman, Dani Lucas‐Barbosa, Kamrul Hassan, Erik H. Poelman

**Affiliations:** ^1^ Laboratory of Entomology Wageningen University Wageningen The Netherlands; ^2^ Present address: Bio‐communication & Ecology ETH Zürich Schmelzbergstrasse 9 8092 Zürich Switzerland; ^3^ Present address: Hawkesbury Institute for the Environment Western Sydney University Locked Bag 1797 Penrith NSW 2751 Australia

**Keywords:** florivores, herbivore‐induced plant responses, indirect interactions, ontogenetic trajectories, ontogenetic variation, plant defence, plant reproduction, pollinators

## Abstract

1. Plants show ontogenetic variation in growth–defence strategies to maximize reproductive output within a community context. Most work on plant ontogenetic variation in growth–defence trade‐offs has focussed on interactions with antagonistic insect herbivores. Plants respond to herbivore attack with phenotypic changes. Despite the knowledge that plant responses to herbivory affect plant mutualistic interactions with pollinators required for reproduction, indirect interactions between herbivores and pollinators have not been included in the evaluation of how ontogenetic growth–defence trajectories affect plant fitness.
2. In a common garden experiment with the annual *Brassica nigra*, we investigated whether exposure to various herbivore species on different plant ontogenetic stages (vegetative, bud or flowering stage) affects plant flowering traits, interactions with flower visitors and results in fitness consequences for the plant.
3. Effects of herbivory on flowering plant traits and interactions with flower visitors depended on plant ontogeny. Plant exposure in the vegetative stage to the caterpillar *Pieris brassicae* and aphid *Brevicoryne brassicae* led to reduced flowering time and flower production, and resulted in reduced pollinator attraction, pollen beetle colonization, total seed production and seed weight. When plants had buds, infestation by most herbivore species tested reduced flower production and pollen beetle colonization. Pollinator attraction was either increased or reduced. Plants infested in the flowering stage with *P. brassicae* or *Lipaphis erysimi* flowered longer, while infestation by any of the herbivore species tested increased the number of flower visits by pollinators.
4. Our results show that the outcome of herbivore–flower visitor interactions in *B. nigra* is specific for the combination of herbivore species and plant ontogenetic stage. Consequences of herbivory for flowering traits and reproductive output were strongest when plants were attacked early in life. Such differences in selection pressures imposed by herbivores to specific plant ontogenetic stages may drive the evolution of distinct ontogenetic trajectories in growth–defence–reproduction strategies and include indirect interactions between herbivores and flower visitors.
5. *Synthesis*. Plant ontogeny can define the direct and indirect consequences of herbivory. Our study shows that the ontogenetic stage of plant individuals determined the effects of herbivory on plant flowering traits, interactions with flower visitors and plant fitness.

## Graphical abstract

Plants show ontogenetic variation in growth–defence strategies to maximize reproductive output within a community context. Our study shows that the ontogenetic stage of plant individuals determined the effects of herbivory on plant flowering traits, interactions with flower visitors and plant fitness. Hence, studies on plant evolution need to consider ecologically relevant timing of herbivory.

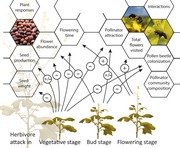

## 1 INTRODUCTION

Interactions between species are the foundations of ecological communities. Ontogenetic stages affect the role that individuals of a species play within a community (Miller & Rudolf, 2011; Nakazawa, 2015). For example, plants gradually develop from seedling through pre‐reproductive and reproductive stages, to eventually senesce and die (Boege & Marquis, 2005). During each of these stages, plants interact with different community members such as antagonistic herbivores, competing plants or beneficial pollinators. To maximize their fitness, plants may display ontogenetic variation in: resistance to herbivores (Barton & Koricheva, 2010; Boege & Marquis, 2005), investment in growth to outcompete neighbours for light (Tonnabel, David, & Pannell, 2017; Zhang, Zhou, Huang, Japhet, & Sun, 2008) or investment in the recruitment of natural enemies of herbivores to reduce the impact of herbivore attack (Quintero, Barton, & Boege, 2013; Quintero, Lampert, & Bowers, 2014).

Plants are part of dynamic communities that constantly shift the cost‐benefit balance between plant growth and defence. Distinct ontogenetic trajectories in growth–defence strategies may allow plants to optimize responses to such shifts (Barton & Boege, 2017). However, ontogenetic growth–defence trajectories come with important implications. First, investment in one component may energetically trade‐off against other components of the trajectory. Second, investment in one component may alter plant interactions with other community members (de Vries, Evers, & Poelman, 2017; Dutton, Luo, Cembrowski, Shore, & Frederickson, 2016; Lucas‐Barbosa, 2016; Villamil, 2017). For example, large plants may be more apparent to herbivores as a result of increased investment in growth induced by competition for light (de Vries, Evers, Dicke, & Poelman, 2019). Plant defence responses to herbivory can affect plant reproduction by changes in flowering time, flower abundance and plant interactions with flower visitors (Rusman, Lucas‐Barbosa, Poelman, & Dicke, 2019). Surprisingly, despite the fact that pollinators are essential for the successful reproduction of most plant species, these have seldom been included in theoretical frameworks of growth–defence trade‐offs through plant ontogeny (but see Villamil, 2017).

Herbivores may affect flower visitors in various ways. For example, herbivores may directly affect flower visitation by physically repelling pollinators (Canela & Sazima, 2003), or indirectly by removing flower biomass that makes plants less attractive to flower visitors (Sõber, Moora, & Teder, 2010). The effects of herbivores on pollinators may be more extensive through indirect interactions mediated by herbivore‐induced plant responses. Defence and reproductive traits are physiologically linked via multiple mechanisms such as resource trade‐offs, shared phytohormonal signalling pathways, shared genetic and biochemical pathways (Jacobsen & Raguso, 2018; Lucas‐Barbosa, 2016; Rusman, Lucas‐Barbosa, et al., 2019). In addition, individual traits can have both defensive and reproductive functions. As a consequence, plant defensive responses induced by herbivores affect flower traits. For example, flowering plants under attack by caterpillars change floral volatile emission to attract natural enemies of the caterpillars. These changes at the same time reduce the attraction of bumblebees that use floral volatiles during foraging (Schiestl, Kirk, Bigler, Cozzolino, & Desurmont, 2014). Indeed, herbivore‐induced changes in expression of flower traits affect the visitation of mutualistic and antagonistic flower visitors (McArt, Halitschke, Salminen, & Thaler, 2013; Rusman, Poelman, Nowrin, Polder, & Lucas‐Barbosa, 2019; Stam, Dicke, & Poelman, 2018). Because flower visitors directly interact with the reproductive organs of the plant, herbivore–flower visitor interactions may come with important plant fitness consequences (Chautá, Whitehead, Amaya‐Márquez, & Poveda, 2017; Moreira, Castagneyrol, Abdala‐Roberts, & Traveset, 2019; Rusman, Lucas‐Barbosa, & Poelman, 2018).

Plant ontogeny is a crucial factor that may determines the outcome and fitness consequences of herbivore–flower visitor interactions. The costs of herbivory vary over plant ontogeny, as well as plant responses to herbivory (Boege, Dirzo, Siemens, & Brown, 2007; Brütting et al., 2017; Diezel, Allmann, & Baldwin, 2011; Rostás & Eggert, 2008). For example, juvenile/vegetative plants may experience higher costs of herbivory compared to flowering plants due to the consumption of important photosynthetic tissues. Juvenile/vegetative plants may respond to herbivore attack with expensive resistance traits to protect those valuable tissues, whereas flowering plants may rely on constitutive defences and/or tolerance mechanisms (Boege et al., 2007; Boege & Marquis, 2005; Lucas‐Barbosa et al., 2017; Ochoa‐López, Villamil, Zedillo‐Avelleyra, & Boege, 2015). Hence, attack early in plant life might reduce resource availability for reproduction. Resource trade‐offs between herbivore resistance and reproduction become apparent in expensive flower traits, such as flowering time, flower abundance, nectar and pollen production (Barber et al., 2015; Poveda, Steffan‐Dewenter, Scheu, & Tscharntke, 2005; Quesada, Bollman, & Stephenson, 1995; Strauss, Conner, & Rush, 1996). Indeed, plants attacked early in development produced smaller inflorescences as compared with non‐damaged plants and plants attacked late in development (Hoffmeister, Wittköpper, & Junker, 2016). In addition to resource‐based mechanisms, flowering plants change flower traits as part of their defensive response induced by herbivory (Rusman, Lucas‐Barbosa, et al., 2019). Such changes are apparent in traits that function in both defence and reproduction, such as flower volatiles and colour (Desurmont, Laplanche, Schiestl, & Turlings, 2015; Rusman, Poelman, et al., 2019). For example, flowering turnip plants change floral volatile emission upon herbivore attack to increase the attraction of natural enemies of the herbivores, but these changes reduce pollinator attraction (Schiestl et al., 2014). Herbivore attack early in development might alter flower traits primarily via resource‐based mechanisms, while attack late in development may change flower traits to optimize defensive functions while reducing reproductive functions. Therefore, we expect ontogenetic variation in plant‐mediated herbivore–flower visitor interactions. So far, ontogenetic variation in indirect herbivore–flower visitor interactions and associated fitness consequences have not been investigated.

In this study on the annual plant *Brassica nigra*, we investigated whether exposure of plants at different ontogenetic stages to various herbivore species affects plant flowering traits, interactions with flower visitors and results in fitness consequences for the plant. In a manipulative experiment, we exposed plants in the vegetative, bud or flowering stage to one of six herbivore species. More specifically, we studied whether herbivore attack to plants at these three ontogenetic stages affects (a) plant phenological traits and flower abundance, (b) visitation rates of mutualists (pollinators), (c) abundance of antagonists (florivorous pollen beetles, *Meligethes* spp.) and (d) seed production. By studying ontogenetic variation of effects of herbivory on plant reproduction we aimed at elucidating whether the selection pressures imposed by herbivores vary depending on the plant ontogenetic stage in which the plant was attacked. Such ontogenetic variation in selection pressures potentially drive the evolution of plant defence through their ontogeny (Barton & Boege, 2017; Ochoa‐López, Rebollo, Barton, Fornoni, & Boege, 2018; Poelman & Kessler, 2016).

## 2 MATERIALS AND METHODS

### 2.1 Plant and insects

Black mustard *Brassica nigra* L. is an annual plant belonging to the cabbage and mustards family (Brassicaceae). Plants grow often in high‐density stands on open river banks and floodplains, and as early successional species in disturbed areas. This species is considered to be an obligate outcrossing species (Conner & Neumeier, 1995), with a generalized pollination system (Lucas‐Barbosa, van Loon, Gols, Beek, & Dicke, 2013; Rusman et al., 2018). Plants flower for several weeks in which hundreds of small yellow flowers with four petals are produced. New flowers open daily, with a relatively short longevity of 3–5 days. Flowers are hermaphroditic, that is contain both male and female structures.

We used seeds of a Black mustard accession (CGN06619) that originates from the Centre for Genetic Resources (CGN). Seeds were propagated by open field pollination and germinated in trays. One‐week‐old plants were transplanted to and cultivated in pots (Ø 17 cm; 2 L) under greenhouse conditions (23 ± 2°C, 50%–70% r.h., L16:D8). Pots were filled with potting soil (Lentse potgrond) and sand in a 1:1 volume ratio. When plants were 2‐week‐old they were transferred to an outside area protected by an insect screen. Three‐week‐old plants were transplanted into the field.

Black mustard plants are colonized by a diverse herbivore community, comprising more than 30 species, of which most are specialist herbivores. We exposed plants to six herbivore species from three herbivore functional groups (HFGs), namely two chewing herbivores (the caterpillar *Pieris brassicae* and sawfly *Athalia rosae*), two sap‐feeding herbivores (the aphids *Brevicoryne brassicae* and *Lipaphis erysimi*) and two root‐feeding herbivores (the cabbage root fly *Delia radicum* and nematode *Heterodera schachtii*). In the field, the above‐ground herbivores colonize plants throughout the season when plants are still vegetative seedlings till large flowering plants (E. Poelman and Q. Rusman, pers. obs.). This information is largely lacking for the root herbivores. Natural colonization densities for above‐ground herbivores range between 1 and 30 chewing herbivore larvae or adult aphids per plant (E. Poelman and Q. Rusman, pers. obs.), for the cabbage root fly five to nine larvae per plant (Soler, 2009) and for nematodes about 1,000 eggs and juveniles per 100 g soil (H. Helder, pers. comm.).

The caterpillar *P. brassicae*, the sawfly *A. rosae* and the aphids *B. brassicae* and *L. erysimi* are routinely reared in the Laboratory of Entomology (Wageningen University) under greenhouse conditions (22 ± 1°C, 50%–70% r.h., L16:D8). *Pieris brassicae* and *B. brassicae* were reared on Brussels sprouts *Brassica oleracea* variety *gemmifera* cultivar Cyrus; *A. rosae* and *L. erysimi* were reared on *Raphanus sativus*. Larvae of the cabbage root fly *D. radicum* were reared on turnips *Brassica rapa* or rutabaga (*Brassica napus*) in a climate cabinet (22 ± 1°C, 50%–70% r.h., L16:D8). Nematodes were reared on rapeseed *B. napus* cultivar Jennifer. Cysts were hatched in the laboratory using a 3 mM ZnCl_2_ solution (Rusman et al., 2018). After hatching, nematodes were flushed out of the hatching sieve using tap water, and solutions containing about 1,000 nematodes (J2 stage) in 4 ml of water were used to infest plants. The caterpillar *P. brassicae*, and the aphids *B. brassicae* and *L. erysimi* were originated from the surroundings of Wageningen (The Netherlands), while the sawfly *A. rosae* originated from surroundings of Würzburg (Bavaria, Germany), the cabbage root fly *D. radicum* from Zeewolde (The Netherlands) and cysts of the nematode *H. schachtii* from the rearing of the Institute for Rational Sugar production (IRS; Bergen op Zoom, The Netherlands). The population used was IRS 07‐01‐04.02 and originated from Woensdrecht, The Netherlands.

### 2.2 Common garden experiment—Field design

A common garden experiment was designed to investigate whether herbivore infestation of plants at different developmental stages affected flowering traits (number of inflorescences and phenological traits), flower visitors (mutualistic pollinators and antagonistic pollen beetles, *Meligethes* spp.) and plant seed production. We planted 160 plots of *B. nigra* in a field of the experimental farm of Wageningen University, The Netherlands (51°59′N, 5°39′E). Plots were organized in 10 rows and 16 columns, and each plot was composed of five plants—one central plant and four plants surrounding the central plant—at a distance of 20 cm. Distance between central plants of neighbouring plots was 1.5 m. Each day 24 plots were planted, except for day 7, when we planted 16 plots. Plots of columns 1–8 were planted between days 1 and 4, column 5 was kept empty, and plants of columns 9–16 were planted between days 4 and 7. Treatments were randomly assigned over plots using a Latin square design, that is each combination of herbivore species and plant developmental stage never occurred twice in the same row or column. Treatments were equally divided over the planting dates and replicated eight times. The experiment was performed from the beginning of May to the end of August (2016).

### 2.3 Plant treatments

**Figure 1:**
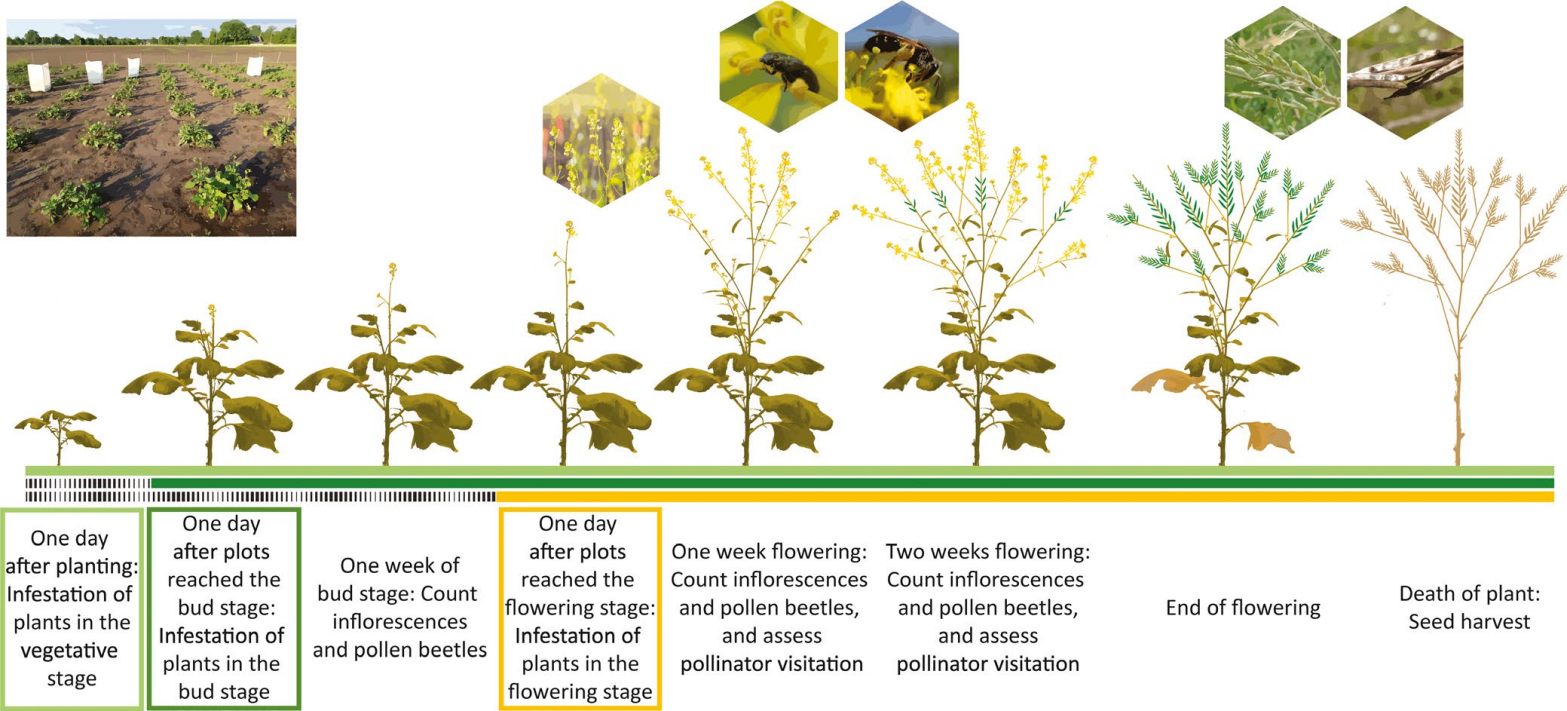
Schematic representation of the timeline of the experiment. All *Brassica nigra* plants were planted at the same time in the field. Plants exposed to herbivores in the vegetative stage (light green bar) were infested 1 day after planting. Plants exposed to herbivores in the bud stage (dark green bar) were infested 1 day after plots had reached the bud stage. Plants exposed to herbivores in the flowering stage (yellow bar) were infested 1 day after plots had reached the flowering stage [Colour figure can be viewed at http://wileyonlinelibrary.com]

Plants were infested with herbivores at different developmental stages, either in the vegetative, bud or flowering stage (Figure 1). Plots in the vegetative stage, growth stage 14–17 (based on *B. napus*; Meier, 2001) were infested one day after planting. Plots in the bud or flowering stage were infested one day after three of the five plants of a plot had reached the bud or flowering stage, including the central plant. We considered that a plant had reached the bud stage when buds of the first flowering stalk rose above the leaves (growth stage 53; Meier, 2001). Plants were considered flowering when the first flower opened (growth stage 60; Meier, 2001). We placed a mesh tent (95 l × 95 w × 190 hr/cm) for 24 hr over each plot for infestation, to provide the necessary time for the herbivores to settle on the plants. Uninfested control plots were also covered with a mesh tent for 24 hr right after planting, when all five plants were still in the vegetative stage.

We infested *B. nigra* plants by placing 10 first instar‐chewing herbivores or 10 adult sap‐feeding herbivores on two true leaves (five per leaf), or by placing 10 first instar *D. radicum* larvae at the base of the plant stem. To infest a plant with nematodes, 4 ml of solution containing about 1,000 nematodes was added in four holes (1 ml per hole) around the main stem of the plant (Rusman et al., 2018). Such holes were approximately 10 cm deep with a diameter of 0.5 cm, and were made at 2 cm from the stem of each of the five plants. For all insect herbivores we used densities that were representative for intermediate natural infestation densities.

Six days after infestation we monitored the survival of all above‐ground herbivore species for each plant. Plants were re‐infested with five second instar‐chewing larvae or five adult‐sucking herbivores when two or fewer herbivores were recorded. At the end of the experiment, root samples were taken from approximately 80 plants and checked for damage by *D. radicum* or nematode presence.

### 2.4 Effect of herbivore infestation and plant ontogeny on flower abundance and phenological traits

To investigate if flower abundance and several phenological traits of *B. nigra* were influenced by herbivore exposure at different plant developmental stages, we recorded flower abundance and followed plant development of infested and uninfested plants. Flower abundance was assessed by counting all inflorescences of each plant at three time points (Figure 1): (a) 7–9 days after plants had entered the bud stage, and at (b) 7–9 and (c) 14–16 days after plants had started flowering. Plant phenological traits were assessed by monitoring plant development daily. We recorded the start of the bud stage, the start of the flowering stage and the end of flowering. Plants were considered finished with flowering when all buds and flowers were gone, and only developing and ripe siliques remained on the flower stalks. We calculated the duration of the bud stage by subtracting the number of days needed to reach the bud stage from the number of days needed to reach the flowering stage. We calculated the duration of the flowering stage by subtracting the number of days needed to reach the flowering stage from the number of days to the termination of flowering.

### 2.5 Effects of herbivore infestation and plant ontogeny on floral mutualists and antagonists

To investigate if pollinator visitation to *B. nigra* flowers was influenced by herbivore exposure of plants at different developmental stages, we recorded pollinator behaviour in plots of infested and uninfested plants at two time points during the following flowering stage: (a) between 7 and 9 days and (b) between 14 and 16 days (Figure 1). Pollinator visitation to a plot was monitored for 10 min. When a pollinator entered the plot and had contact with a flower, identity of the pollinator, number of flowers visited and time spent per flower were recorded (Rusman et al., 2018). The identity of other pollinators that visited the plot during the observation of a pollinator was recorded as well. If the same pollinator individual returned to the plot under observation after having visited a different plot, we scored that visit as a new visit (Rusman et al., 2018). Pollinators were placed in one of the following six pollinator groups: honeybees (HB) *Apis mellifera*, bumblebees *Bombus terrestris*, *Bo. lapidarius*, *Bo. pascuorum* and other *Bombus* spp., syrphid flies (SF; several *Eristalis* spp. and several other syrphid species), solitary bees (several *Andrena* and *Lasioglossum* species but also other Apidae excluding *Bombus* spp.), other flies (non‐syrphid Diptera) and butterflies (*Pieris* spp. and other Lepidoptera). Recordings were done using a handheld computer (Psion Workabout Pro^tm^ 3) programmed with The Observer XT software (version 10, Noldus Information Technology, Wageningen, The Netherlands). Recordings were done during the day (between 9 a.m. and 1 p.m., or 2 p.m. and 5 p.m.) and only when weather conditions were favourable for pollinator activity (15–30°C and wind speed ≤6 m/s; Rusman et al., 2018).

To investigate if pollen beetle (*Meligethes* spp.) colonization was influenced by plant exposure to herbivores in different plant developmental stages, we monitored pollen beetle abundance on plots of infested and uninfested plants. We counted the number of adult pollen beetles on each plant of a given plot at the same three time points we assessed flower abundance (Figure 1). Recordings were done during the day (2–6 p.m.) and only when weather conditions were favourable for pollen beetle activity (15–30°C and wind speed ≤6 m/s; Rusman et al., 2018).

### 2.6 Effects of herbivore infestation at different plant ontogenetic stages on plant seed production

To investigate if life‐time seed production was influenced by herbivory during different plant developmental stages, we assessed seed number and biomass of the plants. We harvested seeds of three plants for each plot; the central plant and two side plants (randomly selected and not adjacent to each other). First harvesting date for each plant was selected before the first siliques would lose their seeds (Rusman et al., 2018). At first harvest, we collected all ripe siliques and left immature siliques and flowers on the plant. Then, plants were checked weekly and siliques harvested when ripe. Siliques were stored in paper bags in a dry storage room until seeds were manually extracted from the siliques. We calculated total number of seeds per plant by weighing 100 seeds, and the total weight of seeds harvested per plant (Rusman et al., 2018). We estimated the total number of seeds by dividing total seed weight by the weight of 100 seeds and multiplied the result by 100. The weight of one seed was estimated by dividing the weight of 100 seeds by 100.

### 2.7 Statistical analysis

For count data such as the number of insects, flowers, days and seeds, we used GL(M)M with a Poisson distribution and a log link function, or negative binomial distribution with a log link function to correct for overdispersion. We ran two models: The first model included herbivore treatment and plant developmental stage and their interactions as fixed factors. In the case of numbers of insects or flowers, this model was run for each time point separately because (a) not all time points included all plant developmental stages and (b) we were interested in species‐specific effects of herbivory on plant flowering traits and interactions with flower visitors, and exploring patterns of higher functional levels (i.e. plant ontogeny) rather than changes over time. Uninfested control plants were excluded from these analyses, because they could not be assigned to any plant developmental stage. The second model included herbivore treatment nested in HFG, time point (except for phenological traits or seeds) and the interaction between herbivore treatment and time point as fixed factors. This model was run for each plant developmental stage separately. Interactions were removed from the models if they were statistically non‐significant (*p > *.05). For post hoc analyses we used Tukey's post hoc tests. Random factors were selected using a backward approach; all random factors such as day (not for flowering traits or seeds), time (morning vs. afternoon; only for pollinators), plot (not for pollinators), plant position (not for pollinators), day*treatment (not for flowering traits or seeds) were added to the model and removed if they explained <3% of the variation or were statistically non‐significant (*p* > .05). We used the lme4 (Bates, Maechler, Bolker, & Walker, 2015), multcomp (Hothorn, Bretz, & Westfall, 2008) and lsmeans (Lenth, 2016) packages for these analyses. For continuous data such as time spent per visit and per flower by pollinators, we used linear (mixed) models with a Gaussian distribution and identity link function or a Gamma distribution with a log link function if the data did not follow a normal distribution. The same fixed factors, random factor selection approach and software packages as for count data were used. We analysed pollinator community composition by comparing the pollinator community composition of infested and uninfested plots with a chi‐squared test (Rusman et al., 2018). Expected pollinator community composition was calculated by summing pollinators within each pollinator group for all plots and dividing this number by the total number of pollinators. This results in an expected percentage for each pollinator group. This percentage was then multiplied by the total number of pollinators recorded for infested or uninfested plots (Rusman et al., 2018). We calculated expected community composition based on pollinators visiting all treatment groups because the pollinator community distributes over the different treatments including the uninfested plots based on pollinator preference in the choice situation, for example the community composition of the uninfested plots is affected by the presence of the infested plots (Rusman et al., 2018). If pollinator community composition was explained by plant exposure to herbivores, pairwise comparisons among all herbivores within one plant developmental stage were performed, and pairwise comparisons among the three plant developmental stages for each herbivore species were performed using chi‐squared tests. To correct for multiple tests of pairwise comparisons, we adjusted the *p‐*values using the false discovery rate correction. We used the fifer package for these analyses (Fife, 2014). In addition, to assess which pollinator groups contributed to differences between herbivore species and plant developmental stages, we calculated the standardized residuals for each pollinator group in each treatment (Rusman et al., 2018). We used a threshold value of ±2, for example residual values higher than +2 or lower than −2 indicate a significant contribution of that pollinator group to the differences in pollinator community composition (Sharpe, 2015). Pollinator groups which composed <1% of the community were excluded from the analysis, for example other flies (0.3%), butterflies (0.04%). For correlations between the number of inflorescences and insects, we computed the correlation coefficient *r* using the Pearson or Kendall method, depending on the distribution of the data. For correlations between the number of inflorescences and pollinators, we averaged the number of inflorescences per plot. Correlation graphs were made using the ggpubr package (Kassambara, 2018). All analyses were carried out in r (version 3.4.3 × 64, 2017, The R Foundation for Statistical Computing Platform).

## 3 RESULTS

Plant ontogeny determined the effects of herbivory on flowering plant traits, interactions with flower visitors and plant seed production. Below we provide details for the effects of herbivory at different plant ontogenetic stages on flower abundance, pollinator attraction, pollen beetle colonization and plant reproduction. Effects on plant phenological traits, pollinator community composition, pollinator visitation and correlations between numbers of pollinators/pollen beetles and flowers are described in the Supporting Information.

### 3.1 Effects of herbivore infestation and plant ontogeny on flower abundance

**Figure 2:**
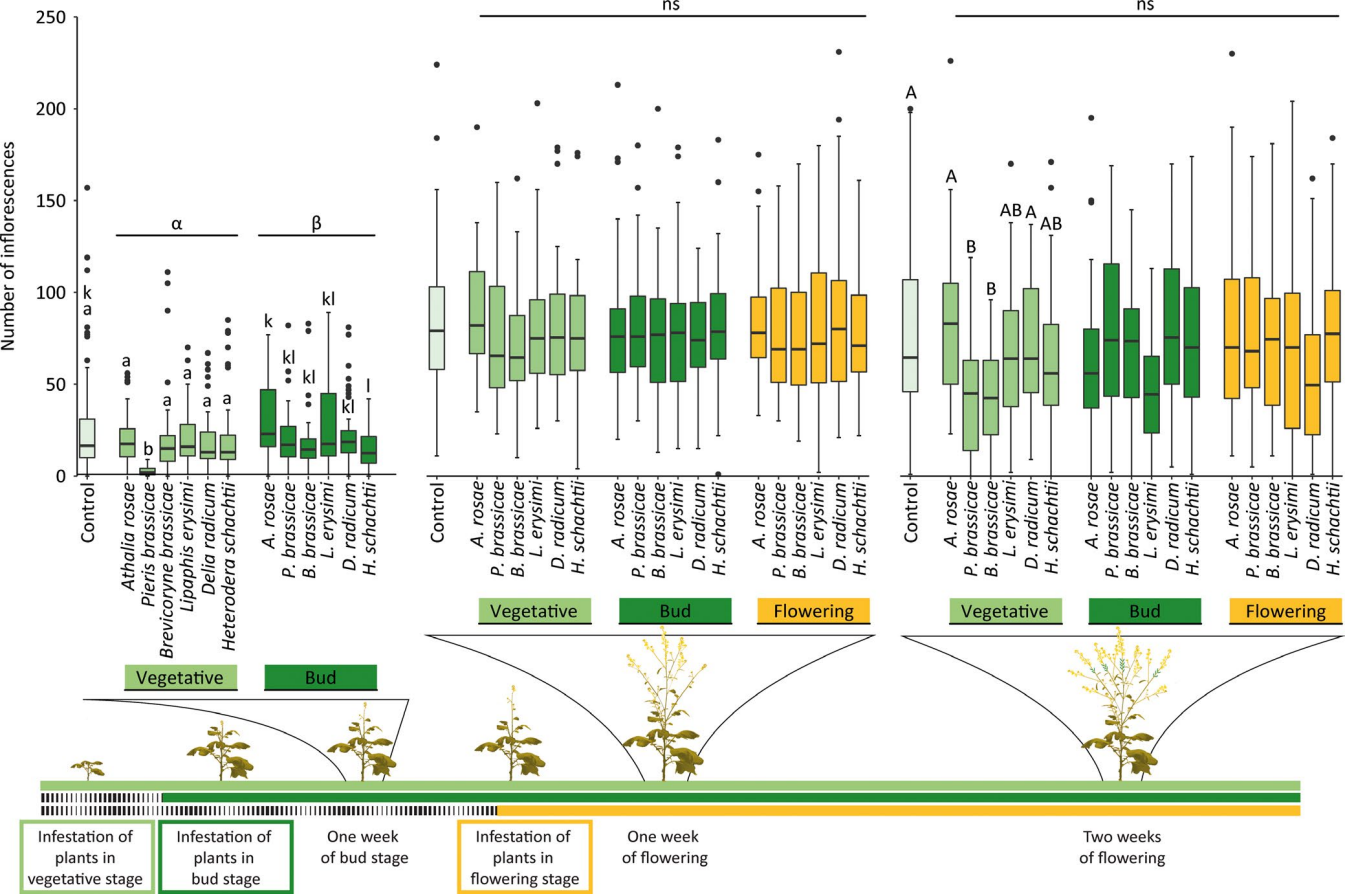
Number of inflorescences of uninfested *Brassica nigra* plants and plants infested with herbivores at different plant ontogenetic stages. Boxplots show median (line), first and third quartiles, minimum and maximum. Outliers (1.5 times the interquartile range below the first or above the third quartile) are represented by circles. Plants were monitored for the number of inflorescences at three time points: between 7 and 9 days after plants had reached the bud stage, and between 7 and 9 days, and 14 and 16 days after plants had started flowering. Number of replicates per herbivore treatment varied between 28 and 45 plants, and between 76 and 78 for uninfested plants. Letter groups (a,b) and (k,l) above bars indicate significant differences (*p* ≤ .05) between herbivore species within a plant ontogenetic stage based on Tukey's post hoc tests, and small or capital letters were used for different time points. Greek letters above lines indicate significant differences at (*p* ≤ .05) between plant ontogenetic stages based on Tukey's post hoc tests, whereas ns indicates no differences [Colour figure can be viewed at http://wileyonlinelibrary.com]

Plant ontogeny determined the effects of plant exposure to herbivores on the number of flowers produced by *B. nigra*, and these effects were herbivore species‐specific and not associated with the HFG (Figure 2; Tables S3 and S4). One week after plants had started to produce buds, plants exposed to herbivores in the vegetative stage had fewer inflorescence on display compared with plants exposed to herbivores in the bud stage (Tukey's post hoc test, *p* < .001), and this effect was particularly strong for plants exposed to *P. brassicae* caterpillars (Tukey's post hoc test, *p* < .001). Effects of herbivore species on the number of inflorescences were always negative, and observed for plants exposed to *P. brassicae* caterpillars in the vegetative stage and *H. schachtii* nematodes in the bud stage 1 week after plants had started to produce buds (Figure 2). These negative effects were still apparent 2 weeks after plants had started flowering for plants exposed to *P. brassicae* caterpillars in the vegetative stage, and by this time, plants exposed to *B. brassicae* aphids in the vegetative stage also had fewer inflorescences compared to uninfested plants. Effects of *P. brassicae*, *B. brassicae* and *L. erysimi* on the number of inflorescences 2 weeks after plants had started flowering varied depending on the ontogenetic stage in which the plant was attacked (Table S2).

### 3.2 Effects of herbivore infestation and plant ontogeny on floral mutualists—Pollinator attraction

**Figure 3:**
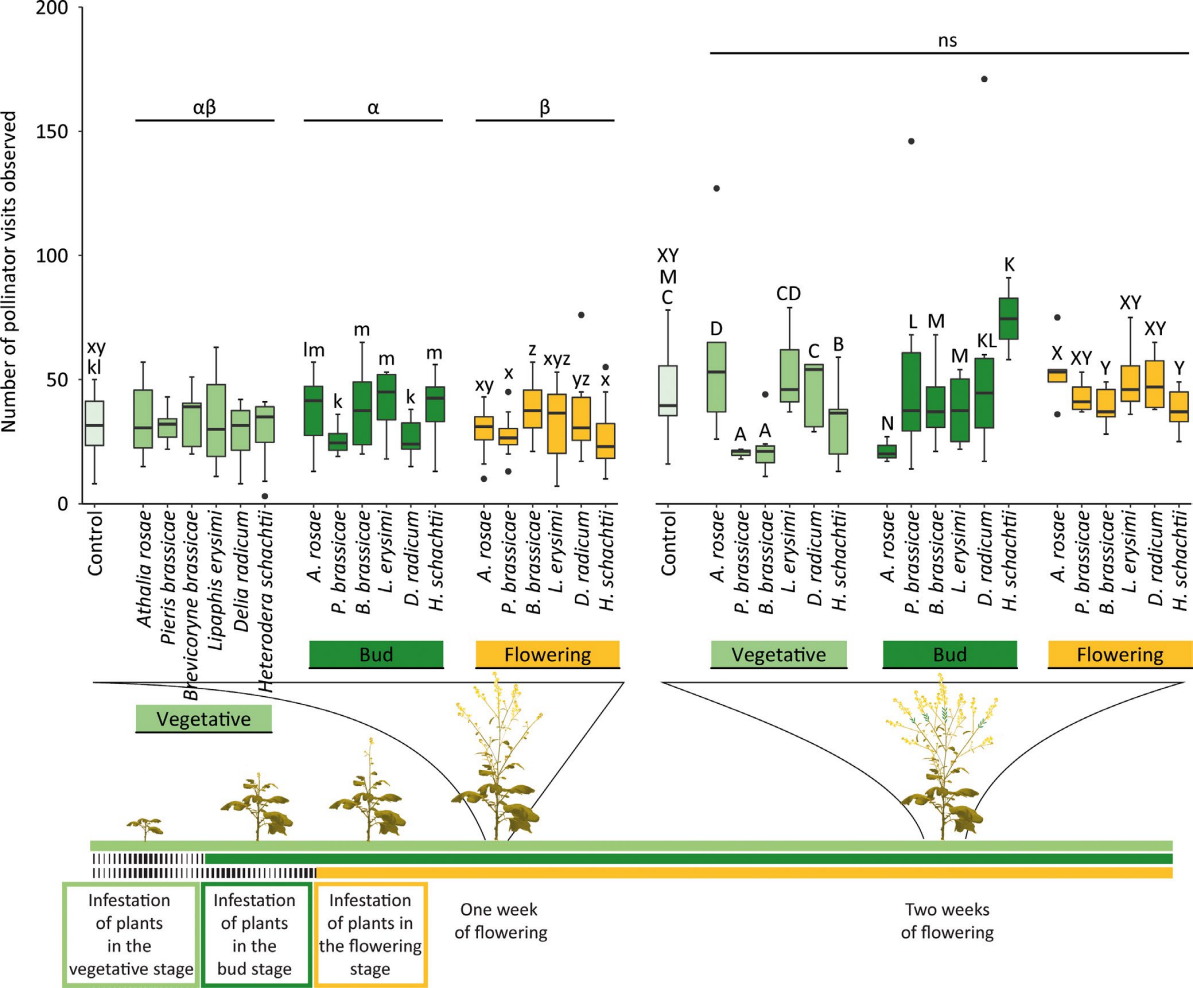
Number of pollinator visits observed on flowers of uninfested plots (control) of *Brassica nigra* plants and on flowers of plots infested by herbivores at different plant ontogenetic stages. Boxplots show median (line), first and third quartiles, minimum and maximum. Outliers (1.5 times the interquartile range below the first or above the third quartile) are represented by circles. Observations lasted for 10 min and were made at two time points: between 7 and 9 days, and 14 and 16 days after plots had started flowerings. For 7–9 days after plots had started flowering, the number of replicates per herbivore treatment varied between 7 and 9, and was 16 for uninfested plants. For 14–16 days after plots had started flowering, the number of replicates per herbivore treatment varied between 2 and 6, and was 10 for uninfested plants. Letters groups (a–d, k–n, x–z) above bars indicate significant differences at (*p* ≤ .05) between herbivore species within a plant ontogenetic stage based on Tukey's post hoc tests, and small or capital letters were used for different time points. Greek letters above lines indicate significant differences (*p* ≤ .05) between plant developmental stages based on Tukey's post hoc tests, whereas ns indicates no differences [Colour figure can be viewed at http://wileyonlinelibrary.com]

One week after plants had started flowering, plots were on average visited by 32 pollinators which visited 89 flowers during the 10‐min observation time, and 2 weeks after plants had started flowering this increased to 44 pollinators and 101 flower visits. Plant ontogeny determined the effects of plant exposure to herbivores on the number of pollinators visiting plots and the number of flowers visited by pollinators, and these effects mostly depended on herbivore identity rather than HFG (Figure 3; Figures S3–S5 and S8–S10; Tables S3 and S4). One week after plants had started flowering, plants exposed to herbivores in the bud stage were visited by a larger number of pollinators, especially honeybees, compared with plants exposed to herbivores in the flowering stage (Tukey's post hoc tests, total pollinators [TP]: *p* = .025, HB: *p* = .034), and effects were particularly strong when plants were exposed to larvae of the sawfly *A. rosae* (Tukey's post hoc tests, TP: *p* = .025, HB: *p* = .034), *L. erysimi* aphids (Tukey's post hoc test, TP: *p* = .007) or *H. schachtii* nematodes (Tukey's post hoc tests, TP: *p* < .001, HB: *p* < .001). However, plants exposed to herbivores in the flowering stage received more syrphid fly visits compared to plants exposed in the vegetative stage (Tukey's post hoc test, *p* = .036). In contrast to the number of pollinator visits, more flowers were visited by all pollinators for plants exposed to herbivores in the flowering stage compared to plants exposed in the bud stage 1 week after the start of flowering (Tukey's post hoc tests, TP: *p* = .006, HB: *p* = .002) and compared to plants exposed in the vegetative stage on both time points (Tukey's post hoc tests, 1 week: TP: *p* < .001, HB: *p* < .001, 2 weeks: TP: *p* < .001, HB: *p* = .005). This was true for four of the six herbivore species (Table S2). The effects of individual herbivore species on the number of TP, honeybees and SF varied with plant ontogeny for both time points (Table S2).

Specific effects of HFG were observed when plants were exposed to herbivory in the flowering stage (total number of flowers visited by HB) 1 week after plants had started flowering (Table S4). Honeybees visited more flowers of plants exposed to sap‐feeding or root herbivores in the flowering stage compared to uninfested plants (Tukey's post hoc tests, *p* = .006 and *p* = .003 respectively). Specific herbivores led to either increased or decreased pollinator visitation when plants were exposed in the vegetative stage (the number of flowers visited by SF), bud stage (the number of TP, HB and SF, and number of flowers visited by TP, HB and SF) or flowering stage (the number of TP and HB, and number of flowers visited by SF) if compared with uninfested plants (Figure 3; Figures S3–S5 and Figures S8–S10; Tables S3 and S4).

### 3.3 Effects of herbivore infestation and plant ontogeny on a floral antagonist

**Figure 4:**
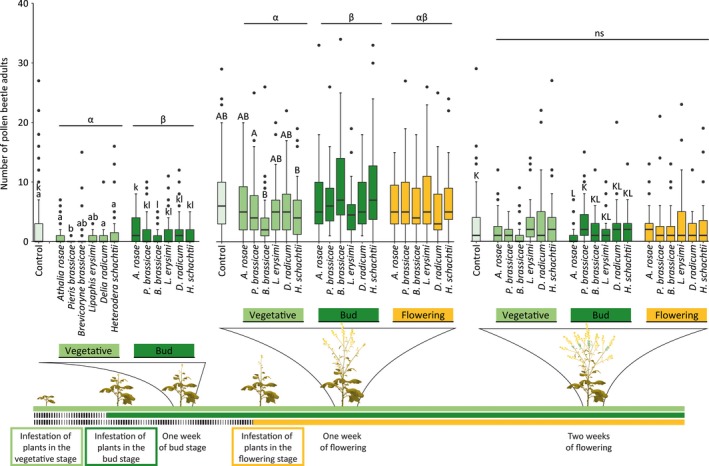
Number of pollen beetle adults (*Meligethes* spp.) observed on flowers of uninfested (control) *Brassica nigra* plants and plants infested by herbivores at different plant ontogenetic stages. Boxplots show median (line), first and third quartiles, minimum and maximum. Outliers (1.5 times the interquartile range below the first or above the third quartile) are represented by circles. Plants were monitored for the number of pollen beetle adults at three time points: between 7 and 9 days after plants had reached the bud stage, and between 7 and 9 days, and 14 and 16 days after plants had started flowering. Number of replicates per herbivore treatment varied between 28 and 45 plants, and between 76 and 78 for uninfested plants. Letters groups (a,b) and (k,l) above bars indicate significant differences at (*p* ≤ .05) between herbivore species within a plant ontogenetic stage based on Tukey's post hoc tests, and small or capital letters were used for different time points. Greek letters above lines indicate significant differences at (*p* ≤ .05) between plant ontogenetic stages based on Tukey's post hoc tests, whereas ns indicates no differences [Colour figure can be viewed at http://wileyonlinelibrary.com]

We observed on average one pollen beetle adult per plant 1 week after plants had started to produce buds; this number increased to an average of seven beetles per plant 1 week after plants had started flowering, and declined to about three beetles per plant 2 weeks after plants had started flowering. Plant ontogeny influenced the effects of plant exposure to herbivores on the number of pollen beetle adults observed per plant, and these effects depended on HFG and herbivore identity (Figure 4; Tables S3 and S4). Plants exposed in the vegetative stage contained fewer adult beetles compared to plants exposed in the bud stage 1 week after plants had started to produce buds (Tukey's post hoc test, *p* = .035) and 1 week after plants had started flowering (Tukey's post hoc test, *p* = .019). Interestingly, this was true for four of the six individual herbivore species 1 week after plants had started to produce buds, and for the other two herbivore species 1 week after plants had started flowering (Table S2).

Compared with uninfested plants, effects of the herbivores *A. rosae*, *P. brassicae* and *B. brassicae* were always negative, and observed for plants exposed in the vegetative stage (1 week after plants had started to produce buds and 1 week after plants had started flowering), and the bud stage (1 week after plants had started to produce buds and 2 weeks after plants had started flowering), but not for plants exposed in the flowering stage (Figure 4; Tables S3 and S4). The effects on plants exposed in the bud stage to herbivores depended on the HFG. One week after plants started to produce buds, plant exposed to chewing herbivores were more heavily colonized by pollen beetles than plants exposed to sap‐feeding herbivores or uninfested plants (Tukey's post hoc tests, *p* = .002 and *p* = .003 respectively). However, 1 week after plants started flowering, plant exposed to chewing and root herbivores had fewer pollen beetles than plants exposed to sap‐feeding herbivores (Tukey's post hoc tests, *p* < .001 and *p* < .001 respectively) or uninfested plants (Tukey's post hoc tests, *p* < .001 and *p* < .001 respectively). Two weeks after plants started flowering, plants exposed to root herbivores had higher numbers of pollen beetles compared to plants exposed to chewing or sap‐feeding herbivores, or uninfested plants (Tukey's post hoc tests, *p* < .001, *p* < .001 and *p* = .016 respectively).

### 3.4 Effects of herbivore infestation and plant ontogeny on plant seed production

**Figure 5:**
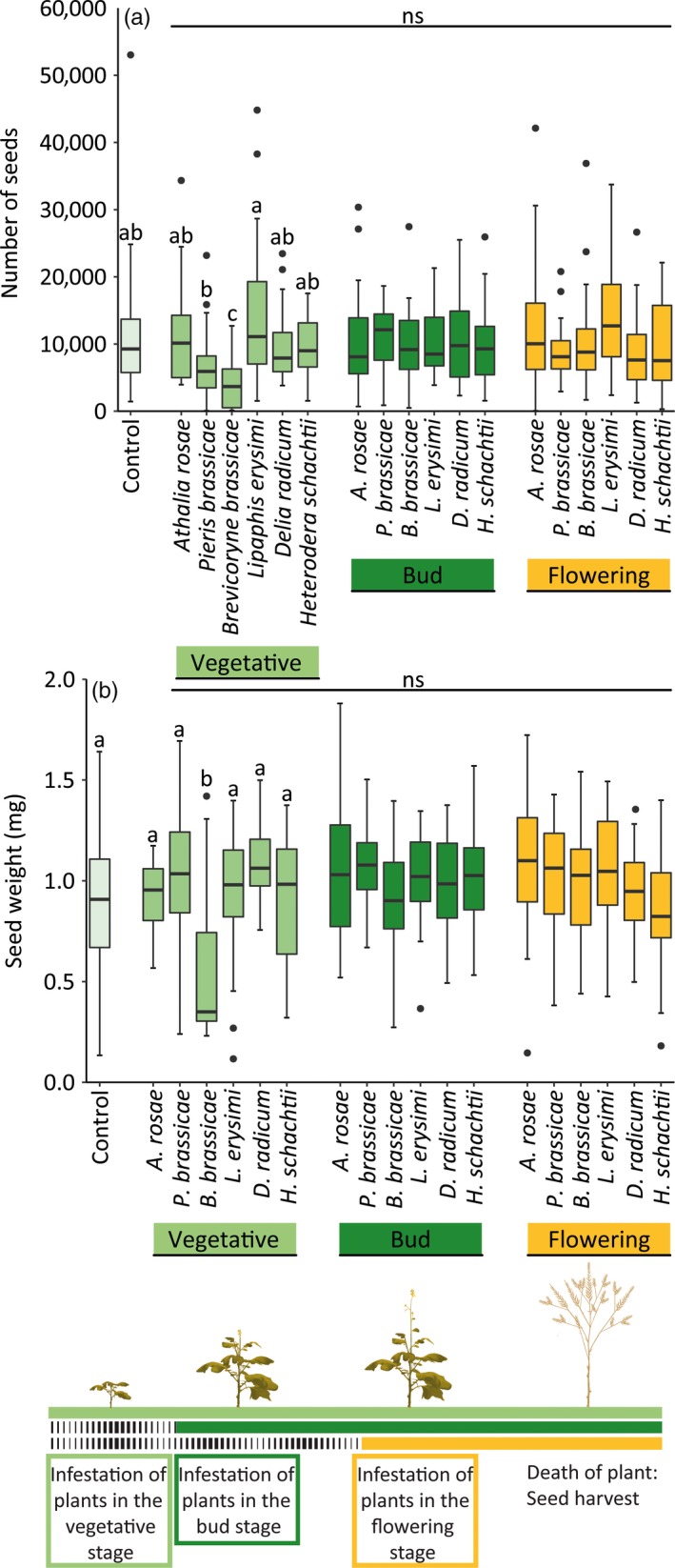
Number of seeds (a) and seed weight (b) of seeds produced by uninfested (control) *Brassica nigra* plants and plants infested by herbivores at different plant ontogenetic stages. Boxplots show median (line), first and third quartiles, minimum and maximum. Outliers (1.5 times the interquartile range below the first or above the third quartile) are represented by circles. Number of seeds and seed weight were averaged for three plants per plot (one central plant and two side plants). The number of replicates per herbivore treatment varied between 43 and 50, and between 88 and 91 for uninfested plants. Letters above bars indicate significant differences at (*p* ≤ .05) between herbivore species within a plant ontogenetic stage based on Tukey's post hoc tests, whereas ns above lines indicates no differences between plant developmental stages [Colour figure can be viewed at http://wileyonlinelibrary.com]

*Brassica nigra* plants produced on average 10,000 seeds, with an average individual seed weight of 1 mg. Overall, plant ontogeny influenced the effects of herbivory on seed numbers and weight (Table S6). Plant ontogeny determined the effects of plant exposure to all herbivores when it comes to side plants, while for central plants and at the plot level these effects were herbivore specific (Figure 5; Figure S20; Table S6). Side plants exposed to herbivores in the vegetative stage produced fewer seeds than plants exposed to herbivores in the flowering stage (Tukey's post hoc test, *p* < .010). This was especially true for plants exposed to *B. brassicae* aphids in the vegetative stage if compared with plants that were exposed to these aphids in the flowering stage (Tukey's post hoc test, *p* < .001) but also in the bud stage (Tukey's post hoc test, *p* < .001). For the average number of seeds produced per plant per plot and seed weight, the effect of individual herbivore species varied with plant ontogeny (Table S2). This was also the case for the number of seeds produced by central plants. Herbivore species‐specific effects on the number of seeds produced were restricted to plants exposed in the vegetative stage (Figure 5a; Figure S20). Plant exposure to *L. erysimi* aphids increased the number of seeds produced, while seed numbers were reduced by exposure to *P. brassicae* caterpillars or *B. brassicae* aphids. Effects of herbivory on seed weight were restricted to plants exposed in the vegetative stage and depended on HFG and herbivore identity (Table S6). Plants exposed to sap‐feeding herbivores produced lighter seeds compared with plants exposed to chewing herbivores, root herbivores or uninfested plants (Tukey's post hoc tests, *p* = .004, *p* < .001 and *p* = .031). Especially the aphid *B. brassicae* reduced the seed weight (Figure 5b).

## 4 DISCUSSION

**Figure 6:**
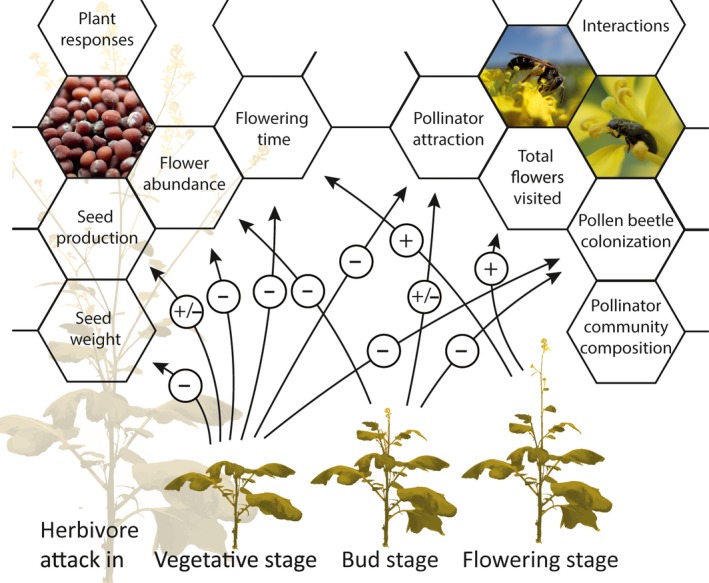
Illustration summarizing the effects of herbivore attack at different plant ontogenetic stages (vegetative, bud, flowering) on flowering traits, seed production and plant interactions with flower visitors. The sign in the circle shows the direction of effect as compared with uninfested plants, where (+) is a positive and (−) a negative effect. No arrow indicates no significant effects as compared with uninfested plants. Effects of herbivory in the vegetative stage were mainly identified for the caterpillar *Pieris brassicae* and aphid *Brevicoryne brassicae*, while effects of herbivory in the bud and flowering stage were spread among the six herbivores used: the sawfly *Athalia rosae*, the caterpillar *P. brassicae*, the aphid *B. brassicae*, the aphid *Lipaphis erysimi*, the cabbage root fly *Delia radicum* and the nematode *Heterodera schachtii* [Colour figure can be viewed at http://wileyonlinelibrary.com]

The findings of our study illustrate that plant ontogeny determines the effects of herbivory on flowering traits, interactions with pollinator mutualists and flower‐feeding antagonists, and plant reproductive output (Figure 6). Effects of herbivory were mostly species‐specific. In few cases—visitation times and flower visits by SF, seed weight—effects depended on the HFG of the herbivore. Plants exposed in the vegetative stage to *P. brassicae* caterpillars or *B. brassicae* aphids resulted in reduced flowering time and flower abundance. Plants infested with these herbivores had reduced pollinator attraction and plant colonization by pollen beetles. Overall, this negatively affected the number and weight of seeds produced. Interestingly, plants exposed in the vegetative stage to *L. erysimi* aphids increased seed production. When plants were exposed in the bud stage to *A. rosae*, *B. brassicae*, *L. erysimi* or *H. schachtii*, herbivory led to reduced flower abundance and pollen beetle colonization. Plants infested with these herbivores received either more or less pollinator visits. Plants exposed to herbivores in the flowering stage received more flower visits by pollinators than plants exposed at the vegetative and bud stages, irrespective of which herbivore we used as inducer. Plants infested in the flowering stage with *P. brassicae* caterpillars or *L. erysimi* aphids flowered longer than uninfested plants or plants infested with the other herbivores. Plant ontogenetic stage defined the effects of herbivory on changes in pollinator flower visitation behaviour, that is increases or decreases in the number of flowers visited, time spent per visit and flower. Taken together, both plant ontogeny and herbivore identity shaped the effects of herbivory on flowering traits, the outcome of indirect interactions with flower visitors, and the consequences for plant fitness.

Our data show that plant ontogeny is a major determinant of indirect interactions between herbivores and flower visitors. Plant‐mediated interactions were specific for the identity of the interaction partners (herbivores and flower visitors) and the direction of the interactions—positive, negative or neutral—varied with plant ontogeny. The resulting indirect interaction web between herbivores and flower visitors appears dynamic and variable over plant ontogeny. Ontogenetic variation in indirect interaction webs is evident from aquatic systems (predatory fish and pelagic and benthic food webs), and systems which include both aquatic and terrestrial components (amphibians or aquatic insects; Miller & Rudolf, 2011; Nakazawa, 2015). However, variation in indirect interaction webs with ontogeny has received limited attention in plant–insect systems (Waltz & Whitham, 1997). Ontogenetic variation in indirect herbivore–flower visitor interactions can be explained by two non‐mutually exclusive mechanisms: different plant responses over plant ontogeny or varying effects of herbivory on plants determined by the timing of herbivore encounter. Indeed, plant responses to herbivory vary with plant ontogeny (Diezel et al., 2011; Ochoa‐López et al., 2015; Rostás & Eggert, 2008), which includes changes in flower traits (Desurmont et al., 2015; Hoffmeister et al., 2016). The timing of events such as herbivory determines the strength of interactions between herbivores and plants, and subsequent effects on flower visitors (Chase, 2003; Stam et al., 2018; Vannette & Fukami, 2014). Herbivory during the vegetative stage of a plant may be costlier as compared to herbivory during the flowering stage, due to increased investments in resistance and loss of important photosynthetic tissues early in life (Boege et al., 2007; de Vries et al., 2019; de Vries, Poelman, Anten, & Evers, 2018). This may lead to reduced investments in flowers because of resource limitations (Barber et al., 2015; Poveda et al., 2005; Quesada et al., 1995; Strauss et al., 1996). Herbivory affects organisms that engage in interactions with the plant under attack soon after the event. Moreover, new interactions established over the rest of the life of the plant are affected until the plant dies (Stam et al., 2018; Stam, Kos, Dicke, & Poelman, 2019; Van Zandt & Agrawal, 2004). The ontogeny of the herbivores themselves likely influences the outcomes of the effects of plant ontogeny on flower‐visitor interactions and plant fitness (Barber, Adler, Theis, Hazzard, & Kiers, 2012; Pineda, Soler, Pastor, Li, & Dicke, 2017). Herbivore species differ in whether their population and damage decrease or increase over time. Caterpillar damage will increase overtime, but their numbers will decrease because of predation and dispersion (Lucas‐Barbosa et al., 2013). Aphids will grow exponentially to become strong nutrient sinks, while nematodes may need time to settle and reproduce before heavy damage is inflicted. Hence, herbivore attack will echo through the indirect interaction web differently depending on the arrival time of a specific herbivore with plant ontogeny.

While herbivore infestation in various plant ontogenetic stages affected plant‐mediated interactions with flower visitors, effects of herbivory on plant flowering traits were most pronounced when herbivores colonized plants early in life, that is during their vegetative stage. Different effects of herbivore infestation on plant traits and on flower visitors suggest the importance of other traits than flower abundance for interactions with flower visitors. Indeed, variation in flower traits such as flower scent and morphology, which we did not assess in this study (but see Rusman, Poelman, et al., 2019), may explain part of the variation in flower‐visitor communities (Kuppler, Höfers, Wiesmann, & Junker, 2016; Soper Gorden & Adler, 2016). Interestingly, plant exposure to herbivores affected the correlation between the number of inflorescences and pollinator mutualists, but not the correlation between the number of inflorescences and antagonistic pollen beetles. This may indicate variable importance of resource quantity and quality for mutualists and antagonists (Cariveau, Irwin, Brody, Garcia‐Mayeya, & Ohe, 2004; Wenninger, Kim, Spiesman, & Gratton, 2016). Floral antagonists may prefer/better assess resource quantity than quality (Althoff, Xiao, Sumoski, & Segraves, 2013; Ekbom & Borg, 1996; Rusman et al., 2018; Wenninger et al., 2016), whereas both may be important for floral mutualists during foraging (Kuppler et al., 2016). Still, we found cases where herbivore exposure did not affect the number of inflorescences but did affect pollen beetle colonization. This could be due to changes in traits that determine flower apparency, such as floral volatiles, which floral antagonists use to locate resources (Jönsson, Rosdahl, & Anderson, 2007; Theis & Adler, 2012). This suggests that antagonist–antagonist interactions are not limited by the dependence of antagonists on resource quantity (Rusman et al., 2018). Herbivory affects flower quantity and quality differently (Hoffmeister et al., 2016; Rusman, Poelman, et al., 2019), and mutualists and antagonists have contrasting effects on plant reproduction (Grass, Bohle, Tscharntke, & Westphal, 2018; Soper Gorden & Adler, 2018). Therefore, variable importance of resource quantity and quality for mutualists and antagonists is likely important for indirect plant fitness consequences of herbivory.

Our data show that fitness consequences of herbivory can be specific to the plant ontogenetic stage that is attacked. Feeding by specific herbivores only affected plant reproduction when plants were colonized by herbivores early in life, while still in the vegetative stage. This indicates that the potential trade‐off between plant growth/reproduction and defence is limited to herbivore attack in specific plant developmental stages (Lucas‐Barbosa, 2016; Lucas‐Barbosa, Loon, & Dicke, 2011). Differences in fitness consequences of herbivory through plant ontogeny can result from direct effects such as allocation costs or developmental constraints (Barton & Boege, 2017), and from indirect effects via plant‐mediated interactions (Lucas‐Barbosa, 2016; Poelman & Kessler, 2016; Strauss, 1997). For annual plants, the main defence strategy early in plant development is resistance, while later in plant development this switches to tolerance (Boege et al., 2007). Increased investment of black mustard in resistance and loss of important photosynthetic tissues due to herbivore damage early in life will be especially expensive (de Vries et al., 2019, 2018). This can explain our observed reduction in flowers, the number of seeds produced and seed weight for plants exposed in the vegetative stage to *P. brassicae* caterpillars or *B. brassicae* aphids. Alternatively, expression of the most effective defence strategy against *P. brassicae* and *B. brassicae* may be limited early in plant development due to developmental constraints (Barton & Boege, 2017; Quintero et al., 2013) or the absence of natural enemies of herbivores early in the season (Gómez‐Marco, Tena, Jaques, & García, 2016; Mira & Bernays, 2002). Herbivory also affects seed production indirectly via interactions with plant‐associated antagonists and mutualists (McArt et al., 2013; Pashalidou et al., 2015; Strauss, Rudgers, Lau, & Irwin, 2002). The reduction in pollinator visitation for plants exposed in the vegetative stage to *P. brassicae* caterpillars or *B. brassicae* aphids likely contributes to the reduced number of seeds produced. Alternatively, plant responses to herbivory may render plants more attractive for subsequent arriving herbivores with associated plant fitness costs (Erwin, Züst, Ali, & Agrawal, 2014), although we did not observe this for specialist pollen beetles (*Meligethes* spp.). Plant interactions with higher trophic levels are also affected by herbivory (Soler, Bezemer, Van Der Putten, Vet, & Harvey, 2005). Plant responses to herbivory may render plants less attractive to natural enemies of other herbivores (Pierre, Dugravot, et al., 2011; de Rijk, Yang, Engel, Dicke, & Poelman, 2016), potentially reducing plant fitness (Hoballah & Turlings, 2001; Pashalidou et al., 2015).

In nature, plants can be colonized by multiple herbivores at the same time or in close sequence. This can lead to complex interactive effects of herbivory on plant‐associated organisms with consequences for plant seed production. For example, plants infested with *H. schachtii* or *Pratylenchus penetrans* nematodes can reduce aphid population growth, while *Meloidogyne hapla* nematodes enhance aphid population growth (Hol, Raaijmakers, Mons, Meyer, & Dam, 2016; van Dam, Wondafrash, Mathur, & Tytgat, 2018). Simultaneous colonization by nematodes and aphids on vegetative plants can thereby negatively or positively influence the effects that aphids have on seed production. Simultaneous herbivory can induce different plant responses as compared to induction by a single herbivore (Pierre, Jansen, et al., 2011; Ponzio, Papazian, Albrectsen, Dicke, & Gols, 2017). This can have consequences for plant‐mediated interactions (Chrétien et al., 2018; Soler et al., 2012; Stam, Chrétien, Dicke, & Poelman, 2017; Stam et al., 2018) and plant seed production (Stam et al., 2019). Ontogenetic variation in networks of indirect plant‐mediated interactions includes these complex interactive effects on plant fitness (Poelman & Kessler, 2016; Rusman et al., 2018; Soper Gorden & Adler, 2018; Stam et al., 2019). Taken together, variation in direct and indirect consequences of herbivory during plant ontogeny likely imposes selection pressures that drive the evolution of plant defence ontogenetic trajectories (Barton & Boege, 2017; Ochoa‐López et al., 2018).

Plant ontogeny is important for direct and indirect consequences of herbivory. Therefore, studies on the evolution of plant defences need to consider ecologically relevant timing of herbivory. Plants can be particularly vulnerable to specific herbivores during certain stages in life, and herbivores that arrive on plants in specific ontogenetic stages can generate particularly strong selection pressures. Plant traits can be effective anti‐herbivore defences during some plant developmental stages, but mediate ecological costs of herbivory in other plant development stages (Barton & Boege, 2017). The adaptive value of traits can therefore only be assessed when considering the complete life cycle of the organisms, and their interactions based on ecologically relevant timing. By determining direct and indirect interactions, ontogeny creates developmental stage‐specific communities which may have profound effects on overall community structure and dynamics (Miller & Rudolf, 2011; Nakazawa, 2015). Moreover, community structure and dynamics may affect trait evolution (Agrawal, Hastings, Johnson, Maron, & Salminen, 2012; Guimarães Jr., Pires, Jordano, Bascompte, & Thompson, 2017; Siepielski & Benkman, 2004; Utsumi, Ando, Roininen, Takahashi, & Ohgushi, 2013), resulting in eco‐evolutionary dynamics driven by ontogenetic variation (Ohgushi, 2016).

## AUTHORS' CONTRIBUTIONS

Q.R., D.L.‐B. and E.H.P. planned and designed the study, interpreted the data and wrote the manuscript; Q.R. and K.H. collected the data; Q.R. analysed the data.

## Data Availability

Data have been deposited in Dryad Digital Repository: https://doi.org/10.5061/dryad.j3tx95x94 (Rusman, Lucas‐Barbosa, Hassan, & Poelman, 2020).
