# Supplementary material for: Plant ontogeny determines strength and associated plant fitness consequences of plant‐mediated interactions between herbivores and flower visitors
Source: J Ecol. 2020 Apr 4;108(3):1046–60. doi: 10.1111/1365-2745.13370 (PMC7217261; doi:10.1111/1365-2745.13370)
Supplement: Supplementary file 1 [file JEC-108-1046-s001.pdf]

Supporting Information

Plant ontogeny determines strength and associated plant fitness consequences of plant-mediated interactions between herbivores and flower visitors

Quint Rusman, Dani Lucas-Barbosa, Kamrul Hassan, and Erik H. Poelman

Corresponding author: quint.rusman@hotmail.com

Effects of herbivore infestation and plant ontogeny on plant phenological traits

Once transplanted into the field, it took plants on average 12 days to reach the bud stage and an additional 10 days to reach the flowering stage. Plants flowered for an average of 29 days. Plant exposure to herbivores affected all plant phenological traits (Fig. S1, Table S1). The ontogenetic stage in which plants were exposed to the herbivores influenced the effects on plant phenological traits: herbivore infestation in the bud stage led to shorter flowering period compared with plants infested in the flowering stage (Tukey's *post hoc* test, P = 0.048). Compared with plants infested in the vegetative stage, herbivore infestation in the bud stage led to a shorter bud stage (Tukey's *post hoc* test, P < 0.001), fewer days to reach the flowering stage (Tukey's *post hoc* test, P < 0.001), and earlier termination of flowering (Tukey's *post hoc* test, P = 0.005). Effects of individual herbivores varied over plant ontogeny, especially for the herbivores *P. brassicae*, *B. brassicae*, and *L. erysimi* (Table S2). Effects of herbivory on phenological traits depended on herbivore functional group (HFG) and herbivore identity if plants were infested in the vegetative stage. Plants exposed in the vegetative stage to sap-feeding or root herbivores reached the bud stage faster than uninfested plants (Tukey's *post hoc* tests, P = 0.032 and P = 0.032 respectively), while plants exposed to chewing herbivores remained in the bud stage longer and took longer to reach the flowering stage than uninfested plants (Tukey's *post hoc* test, P < 0.001 and P = 0.032) and plants exposed to sap-feeding

herbivores (Tukey's *post hoc* test, $P < 0.001$ and $P < 0.001$ respectively) or root herbivores (Tukey's *post hoc* tests, $P < 0.001$ and $P < 0.001$ respectively). Plants exposed in the vegetative stage to chewing herbivores terminated flowering later if compared with plants exposed to sap-feeding or root herbivores (Tukey's *post hoc* test, $P < 0.001$ and $P = 0.007$ respectively). We observed herbivore-species-specific effects of *P. brassicae* and *B. brassicae* for the duration of the bud stage, days till flowering, and termination of flowering (Fig. S1b, c, e). Effects of herbivory on flowering time depended on HFG when plants were infested in the bud stage, whereas herbivore-species-specific effects were observed for plants exposed to herbivory in the vegetative and flowering stage (Fig. S1d, Table S2). Plants exposed in the bud stage to root herbivores flowered longer than plants exposed to sap-feeding herbivores (Tukey's *post hoc* test, $P = 0.002$).

Effects of herbivore infestation and plant ontogeny on floral mutualists – pollinator community

The four most abundant pollinator groups that visited *B. nigra* flowers were honeybees (86%), syrphid flies (11%), bumblebees (2%), and solitary bees (1%). Effects of plant exposure to herbivory on overall pollinator community composition depended on the interaction between herbivore identity and plant ontogeny (Fig. S1, χ^2 test, $\chi^2 = 75.30$, df = 54, $P = 0.025$) rather than solely on herbivore identity (χ^2 test, $\chi^2 = 8.62$, df = 18, $P = 0.968$), plant ontogeny (χ^2 test, $\chi^2 = 10.03$, df = 9, $P = 0.348$), HFG (χ^2 test, $\chi^2 = 2.24$, df = 9, $P = 0.987$), or the interaction between HFG and plant ontogeny (χ^2 test, $\chi^2 = 17.70$, df = 27, $P = 0.912$). Effects of individual herbivore species on pollinator community composition varied over plant ontogeny (Table S2). For example, pollinator community composition differed for plants exposed to *D. radicum* in the bud stage compared to plants exposed to these root-feeding herbivores in the vegetative stage (χ^2 test, $P = 0.004$) or flowering stage (χ^2 test, $P = 0.006$). We found herbivore-species-specific effects on pollinator community composition for plants exposed to herbivores in the

bud stage (χ^2 test, χ^2 = 29.15, df = 18, P = 0.047), but pairwise testing only revealed marginally insignificant differences between herbivores (Fig. S2). All pollinator groups responded to the herbivore treatments and their responses were specific for timing of herbivore attack during plant ontogeny (Fig. S2, Table S5). For instance, solitary bees visited plants infested with the root herbivore *D. radicum* in the bud stage less frequently compared to the expected community, but bumblebees visited these plants more frequently. In contrast, solitary bees visited plants infested in the flowering stage with the root herbivore *D. radicum* more frequently compared to the expected community, but bumblebees visited these plants less frequently (Table S5).

Effects of herbivore infestation and plant ontogeny on floral mutualists – correlations between numbers of pollinators and flowers

The total number of pollinators positively correlated with the number of inflorescences one and two weeks after the start of flowering (Fig. S6 and S7, r = 0.25, t = 3.19, df = 157, P = 0.002 ; r = 0.49, t = 5.08, df = 82, P < 0.001 respectively). In both cases, plant exposure to herbivores affected this correlation (Fig. S6 and S7). One week after plants had started flowering, we found a positive correlation between the number of inflorescences and the total number of pollinators for plants exposed in the flowering stage to herbivores (r = 0.49, t = 3.85, df = 46, P < 0.001), especially to *L. erysimi* aphids (r = 0.79, t = 3.19, df = 6, P = 0.019) and the root herbivore *D. radicum* (r = 0.85, t = 3.95, df = 6, P = 0.008). In contrast, we found a positive correlation for plants exposed to herbivores in the vegetative stage two weeks after the start of flowering stage (r = 0.79, t = 6.75, df = 28, P < 0.001). Due to low number of observations two weeks after the start of flowering, we could only analyse each herbivore species for all three plant ontogenetic stages combined. We found a positive correlation between the number of inflorescences and the total number of pollinators for uninfested plants (r = 0.76, t = 3.29, df = 8, P = 0.011), and

plants exposed to the sawfly *A. rosae* (r = 0.66, t = 2.79, df = 10, $P = 0.019$) or the nematode *H. schachtii* (r = 0.74, t = 3.62, df = 11, $P = 0.004$).

Effects of herbivore infestation and plant ontogeny on floral mutualists – visitation times and flower visits

Plant ontogeny determined the effects of plant exposure to herbivores on pollinator visits: the number of flowers visited per visit (for honeybees (HB) and syrphid flies (SF)), the time spent per flower (for HB and SF), visitation duration (for SF); and effects depended on both herbivore identity and HFG. (Fig. S11 - S16, Tables S3 and S4). One week after plants had started flowering, syrphid flies visited more flowers per visit on plants exposed to herbivores in the flowering stage compared to plants exposed in the bud stage (Tukey's *post hoc* test, $P = 0.044$), and this was especially true for plants exposed to larvae of the sawfly *A. rosae* (Tukey's *post hoc* tests, $P = 0.044$) or the nematode *H. schachtii* (Tukey's *post hoc* tests, $P = 0.039$). Syrphid flies spent more time per flower on plants exposed to herbivores in the bud stage compared to plants exposed in the vegetative stage (Tukey's *post hoc* tests, $P = 0.002$), whereas honeybees spent more time per flower on plants exposed in the vegetative stage compared to plants exposed in the flowering stage (Tukey's *post hoc* tests, $P = 0.033$). In the case of honeybees, this effect was also observed two weeks after plants had started flowering (Tukey's *post hoc* tests, $P = 0.020$), but then honeybees also visited fewer flowers of plants that had been exposed to herbivores in the vegetative stage compared to plants exposed in the flowering stage (Tukey's *post hoc* tests, $P = 0.015$). Effects of individual herbivore species on the visitation behaviour of honeybees and syrphid flies varied over plant ontogeny (Table S2). Specific effects of HFG were observed for plants exposed to herbivory in the vegetative stage (number of flowers visited and time spent per flower by SF), bud stage (number of flowers visited and time spent per plant by SF, time spent per flower by HB), and flowering stage (number of flowers visited by SF).

We detected such changes at the first time point - one week after plants had started flowering (Table S4). Syrphid flies visited more flowers of plants exposed in the vegetative stage to chewing or root herbivores compared with plants exposed to sap-feeding herbivores (Tukey's *post hoc* tests, $P < 0.001$ and $P < 0.001$ respectively) or uninfested plants (Tukey's *post hoc* tests, $P < 0.001$ and $P < 0.001$ respectively). Syrphid flies spent, however, less time on plants infested by chewing herbivores than on uninfested plants if plants had been infested in the vegetative stage (Tukey's *post hoc* tests, $P = 0.033$). When plants were exposed in the bud stage to herbivory, syrphid flies visited more flowers of uninfested plants compared with plants exposed to sap-feeding herbivores (Tukey's *post hoc* test, $P < 0.001$), but less compared with plants exposed to chewing herbivores (Tukey's *post hoc* test, $P = 0.002$). Honeybees spent more time per flower of plants exposed in the bud stage to chewing herbivores than on plants exposed to sap-feeding herbivores (Tukey's *post hoc* test, $P = 0.031$). When flowering plants were exposed to herbivores, syrphid flies visited more flowers of plants exposed to chewing or root herbivores than on plants exposed to sap-feeding herbivores (Tukey's *post hoc* tests, $P < 0.001$ and $P < 0.001$ respectively) or uninfested plants (Tukey's *post hoc* tests, $P < 0.001$ and $P < 0.001$ respectively). Herbivore-species-specific effects were observed for plants exposed in the vegetative stage (visitation duration SF, number of flowers visited by HB and SF), bud stage (number of flowers visited by SF, time spent per flower by HB), and flowering stage (visitation duration, number of flowers visited, and time spent per flower by SF) for one week after plants had started flowering (Fig. S11 - S16, Tables S3 and S4). At two weeks after plants had started flowering, we collected a limited number of observations for syrphid flies and could not analyse the effects of plant exposure to different herbivore species on their visitation behaviour. For honeybees, herbivore-species-specific effects on the number of flowers visited per visit and the time spent per flower were observed for plants exposed in the vegetative and flowering stage,

and for number of flowers visited per visit also for plants exposed in the bud stage (Fig. S12 and S13).

Effects of herbivore infestation and plant ontogeny on a floral antagonist – correlations between numbers of pollen beetles and flowers

The number of pollen beetle adults per plant positively correlated with the number of inflorescences per plant for all three time points: one week after plants had started to produce buds (Fig. S17, $\tau = 0.46$, $z = 32.73$, $P < 0.001$), and one and two weeks after the start of flowering (Fig. S18 and S19, $\tau = 0.45$, $z = 33.87$, $P < 0.001$; $\tau = 0.53$, $z = 37.46$, $P < 0.001$ respectively). We found a positive correlation between the number of inflorescences and the number of pollen beetle adults for all treatments combined, for plants exposed in different ontogenetic stages to herbivores, and for each herbivore species and plant ontogenetic stage combination (Fig. S17 - S19).

Table S1. Output of generalized linear (mixed) models showing the effects of different fixed (herbivore species, plant ontogenetic stage, and herbivore functional group) factors on plant phenological traits. All random factors were initially included in the model, and factors which explained less than 3 percent variation or with a P - value above 0.05 were excluded from the model. Bold values indicate results where $P \leq 0.05$. Italic values indicate results where $P \leq 0.1$.

	Herbivore species (T)			Plant ontogenetic stage (O)			T*O			Herbivore functional group (HFG)			Plot			Plant position		
	df	χ^2	*P*	df	χ^2	*P*	df	χ^2	*P*	df	χ^2	*P*	df	χ^2	*P*	df	χ^2	*P*
Days to reach bud stage	3	3.55	0.314	-	-	-	-	-	-	3	10.14	**0.017**	-	-	-	-	-	-
Length of bud stage	6	66.55	**<0.001**	1	10.19	**0.001**	5	45.15	**<0.001**	-	-	-	-	-	-	-	-	-
Vegetative stage	3	59.15	**<0.001**	-	-	-	-	-	-	3	52.86	**<0.001**	-	-	-	-	-	-
Bud stage	3	3.41	0.333	-	-	-	-	-	-	3	0.933	0.818	-	-	-	-	-	-
Days to reach flowering stage	6	24.06	**<0.001**	1	0.93	0.33	5	34.28	**<0.001**	-	-	-	-	-	-	-	-	-
Vegetative stage	3	24.76	**<0.001**	-	-	-	-	-	-	3	32.58	**<0.001**	-	-	-	-	-	-
Bud stage	3	0.29	0.962	-	-	-	-	-	-	3	1.02	0.796	-	-	-	-	-	-
Length of the flowering stage	6	24.91	**<0.001**	2	5.47	*0.065*	10	39.67	**<0.001**	-	-	-	-	-	-	-	-	-
Vegetative stage	3	22.79	**<0.001**	-	-	-	-	-	-	3	0.34	0.952	-	-	-	-	-	-
Bud stage	3	5.23	0.155	-	-	-	-	-	-	3	13.06	**0.005**	-	-	-	-	-	-
Flowering stage	3	19.60	**<0.001**	-	-	-	-	-	-	3	6.13	0.106	-	-	-	-	-	-
Lifetime	6	20.37	**0.002**	2	5.27	*0.072*	10	36.50	**<0.001**	-	-	-	-	-	-	-	-	-
Vegetative stage	3	27.42	**<0.001**	-	-	-	-	-	-	3	17.96	**<0.001**	-	-	-	-	-	-
Bud stage	3	2.45	0.483	-	-	-	-	-	-	3	5.18	0.159	-	-	-	-	-	-
Flowering stage	3	8.85	**0.031**	-	-	-	-	-	-	3	2.70	0.441	-	-	-	-	-	-

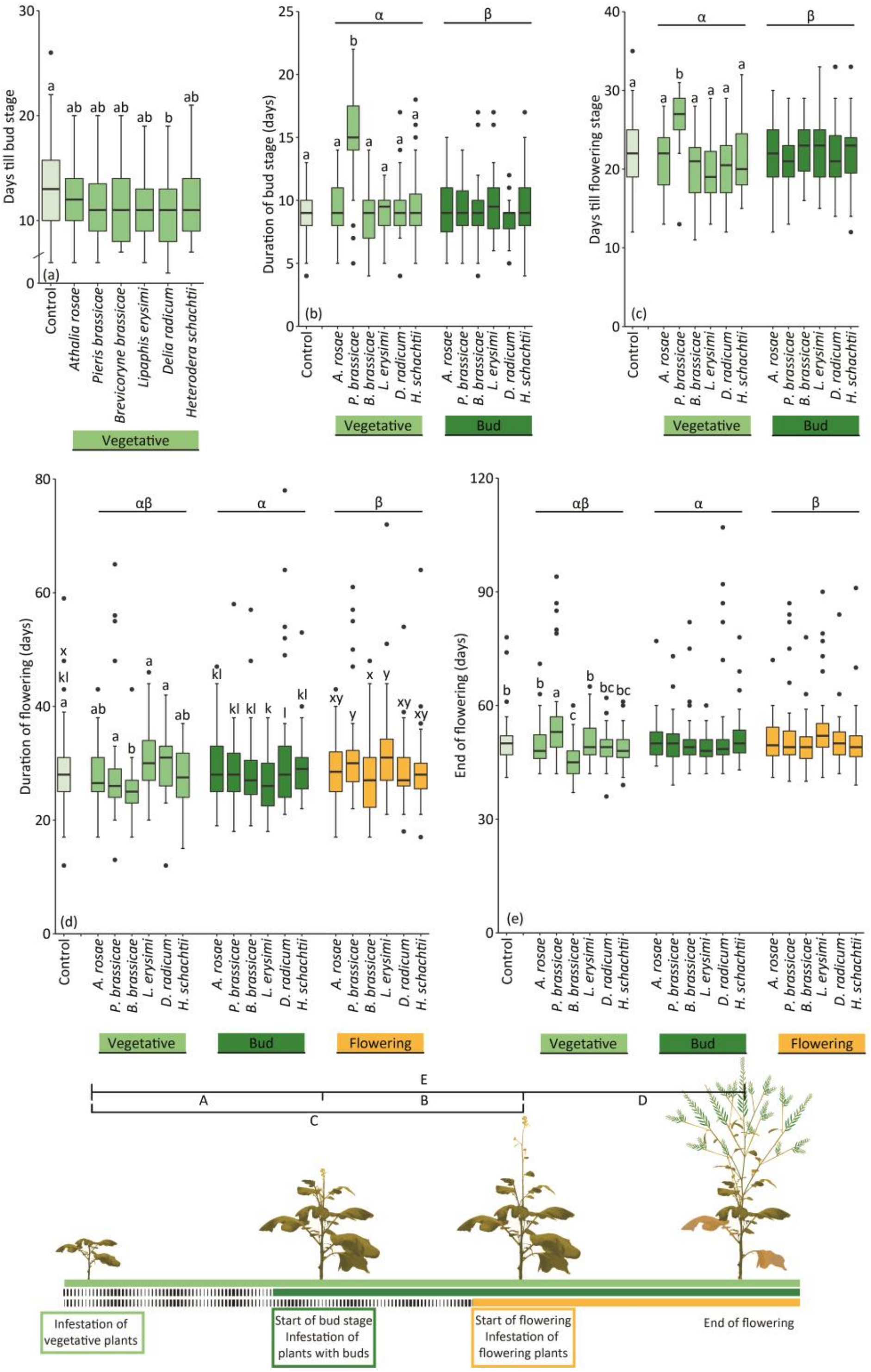

Fig. S1 Phenological traits of uninfested *Brassica nigra* plants and plants infested with one of six herbivore species at different plant ontogenetic stages. We monitored when plants reached the bud stage (a), duration of the bud stage (b), when plants reached the flowering stage (c), duration of flowering (d), and end of flowering (e). Boxplots show median (line), 1st and 3rd quartiles, minimum and maximum. Outliers (1.5 times the interquartile range below the 1st or above the 3rd quartile) are represented by circles. Number of replicates per herbivore treatment varied between 33 and 45 plants, and between 76 and 78 for uninfested plants. Letter groups (a - c, k - l, x - y) above bars indicate significant differences ($P \leq 0.05$) between herbivore species within a plant ontogenetic stage based on Tukey's *post hoc* tests. Greek letters above lines indicate significant differences at ($P \leq 0.05$) between plant ontogenetic stages based on Tukey's *post hoc* tests.

Table S2. Comparisons between *Brassica nigra* plants exposed to herbivores in different plants ontogenetic stages (vegetative, bud, flowering) for various plant traits and insect measurements on different time points. Comparison were made with Tukey's post hoc tests after initial modelling with (generalized) linear (mixed) models. Bold values indicate results where $P \leq 0.05$. Italic values indicate results where $P \leq 0.1$.

	Vegetative-bud	Vegetative-flowering	Bud-flowering	Vegetative-bud	Vegetative-flowering	Bud-flowering
	Length of bud stage			Days to reach the flowering stage		
Pieris brassicae	**<0.001**	-	-	**<0.001**	-	-
Athalia rosae	0.881	-	-	0.653	-	-
Brevicoryne brassicae	0.258	-	-	*0.085*	-	-
Lipaphis erysimi	0.912	-	-	0.609	-	-
Delia radicum	0.576	-	-	**0.048**	-	-
Heterodera schachtii	0.553	-	-	**0.010**	-	-
	Flowering time			Lifetime		
Pieris brassicae	0.917	0.118	**0.048**	**0.005**	0.182	0.353
Athalia rosae	0.822	0.908	0.981	0.686	0.877	0.935
Brevicoryne brassicae	**0.032**	0.142	0.796	**0.005**	**0.016**	0.921
Lipaphis erysimi	**0.002**	0.452	**<0.001**	0.525	*0.056*	**0.003**
Delia radicum	0.203	0.739	**0.033**	*0.056*	0.543	0.417
Heterodera schachtii	0.207	0.443	0.880	0.269	0.492	0.910
	Number of inflorescences 1 week after plants started to produce buds			Number of inflorescences 2 weeks after the start of flowering		
Pieris brassicae	**<0.001**	-	-	**<0.001**	**0.001**	0.981
Athalia rosae	*0.053*	-	-	0.274	0.989	0.317
Brevicoryne brassicae	0.363	-	-	**0.021**	**0.008**	0.901
Lipaphis erysimi	0.105	-	-	*0.092*	0.961	*0.066*
Delia radicum	0.318	-	-	0.932	0.334	0.205
Heterodera schachtii	*0.085*	-	-	0.393	0.278	0.961
	Pollinator community					
Pieris brassicae	0.762	0.762	0.934			
Athalia rosae	0.921	0.921	0.921			
Brevicoryne brassicae	0.276	0.276	0.276			
Lipaphis erysimi	0.284	0.392	0.392			
Delia radicum	**0.004**	0.598	**0.006**			
Heterodera schachtii	*0.095*	*0.095*	0.877			
	Number of pollinators 1 week after the start of flowering			Number of pollinators 2 weeks after the start of flowering		
Pieris brassicae	*0.078*	0.400	0.659	**0.004**	**0.048**	0.601
Athalia rosae	0.516	0.283	**0.025**	**0.002**	0.847	**0.010**
Brevicoryne brassicae	0.213	0.336	0.957	*0.086*	*0.094*	0.987
Lipaphis erysimi	**0.007**	0.992	**0.007**	0.446	0.987	0.575
Delia radicum	0.834	**0.011**	**0.002**	0.454	0.949	0.701
Heterodera schachtii	**0.002**	0.786	**<0.001**	**0.040**	0.857	0.118
	Number of honeybees 1 week after the start of flowering			Number of honeybees 2 weeks after the start of flowering		
Pieris brassicae	**0.007**	*0.097*	0.603	**0.004**	**0.041**	0.638
Athalia rosae	0.403	0.443	**0.034**	**0.003**	0.866	**0.012**
Brevicoryne brassicae	0.164	0.409	0.839	0.167	0.154	0.998
Lipaphis erysimi	0.145	0.988	0.121	0.599	1.000	0.620
Delia radicum	0.954	**0.021**	**0.012**	0.345	0.984	0.498
Heterodera schachtii	**0.021**	0.329	**<0.001**	**0.030**	0.809	0.108

	Number of syrphid flies 1 week after the start of flowering			Number of syrphid flies 2 weeks after the start of flowering		
Pieris brassicae	0.852	0.688	0.951	0.421	0.974	0.110
Athalia rosae	0.992	0.792	0.719	0.205	0.985	0.258
Brevicoryne brassicae	0.461	0.329	0.968	*0.054*	0.118	0.887
Lipaphis erysimi	0.123	0.828	**0.016**	**0.011**	0.258	0.319
Delia radicum	0.596	0.696	0.177	0.306	0.431	0.986
Heterodera schachtii	0.134	0.699	0.411	*0.071*	0.989	*0.054*
	Number of flowers visited by pollinators 1 week after the start of flowering			Number of flowers visited by pollinators 2 weeks after the start of flowering		
Pieris brassicae	0.965	0.331	0.215	0.690	*0.080*	0.228
Athalia rosae	0.242	0.272	**0.005**	0.989	0.584	0.577
Brevicoryne brassicae	0.348	**0.001**	*0.057*	0.923	0.298	0.595
Lipaphis erysimi	0.129	0.974	0.219	0.211	**0.046**	0.789
Delia radicum	0.897	**0.010**	**0.003**	0.318	**0.024**	0.361
Heterodera schachtii	0.322	0.547	0.919	0.292	0.276	0.935
	Number of flowers visited by honeybees 1 week after the start of flowering			Number of flowers visited by honeybees 2 weeks after the start of flowering		
Pieris brassicae	0.141	0.314	**0.002**	0.770	0.159	0.334
Athalia rosae	0.222	0.423	**0.010**	0.988	0.836	0.760
Brevicoryne brassicae	0.494	**0.031**	0.318	0.977	0.673	0.600
Lipaphis erysimi	0.551	0.578	0.999	0.374	0.215	0.945
Delia radicum	0.998	**0.016**	**0.026**	0.194	*0.064*	0.757
Heterodera schachtii	0.285	0.843	0.605	0.619	0.683	0.949
	Number of flowers visited by syrphid flies 1 week after the start of flowering			Visitation time of syrphid flies 1 week after the start of flowering		
Pieris brassicae	0.982	0.150	*0.064*	0.931	0.335	0.128
Athalia rosae	0.460	0.865	0.205	0.959	0.839	0.679
Brevicoryne brassicae	0.999	**0.050**	**0.042**	0.805	**0.036**	0.145
Lipaphis erysimi	0.535	0.801	0.299	0.759	0.794	0.455
Delia radicum	0.993	0.356	0.586	0.971	0.849	0.972
Heterodera schachtii	0.429	0.981	0.503	0.914	0.809	0.968
	Number of flowers visited per visit by syrphid flies 1 week after the start of flowering			Time spent per flower by honeybees 1 week after the start of flowering		
Pieris brassicae	0.647	*0.098*	0.353	*0.058*	**0.001**	0.786
Athalia rosae	0.658	0.466	**0.044**	0.942	0.966	0.998
Brevicoryne brassicae	0.796	0.538	0.233	0.363	0.934	0.195
Lipaphis erysimi	0.122	0.998	0.128	0.708	0.982	0.838
Delia radicum	0.813	0.267	0.148	0.582	0.186	0.684
Heterodera schachtii	*0.053*	0.937	**0.039**	**0.004**	0.641	*0.068*
	Time spent per flower by honeybees 2 weeks after the start of flowering			Number of pollen beetle adults one week after plants started to produce buds		
Pieris brassicae	0.825	0.794	0.996	**<0.001**	-	-
Athalia rosae	0.872	0.290	0.701	**0.048**	-	-
Brevicoryne brassicae	0.942	0.486	0.754	0.476	-	-
Lipaphis erysimi	**0.025**	**0.012**	0.973	**0.016**	-	-
Delia radicum	0.571	0.635	1.000	**0.030**	-	-
Heterodera schachtii	0.992	0.985	1.000	0.181	-	-
	Number of pollen beetle adults 1 week after the start of flowering			Number of pollen beetle adults 2 weeks after the start of flowering		
Pieris brassicae	0.548	0.812	0.893	**0.003**	*0.069*	0.466
Athalia rosae	0.923	0.998	0.896	*0.076*	0.793	**0.012**
Brevicoryne brassicae	**<0.001**	*0.051*	**0.014**	0.484	**0.009**	0.126
Lipaphis erysimi	0.984	0.179	0.143	0.237	0.864	0.115
Delia radicum	0.768	0.899	0.478	0.608	*0.092*	0.495
Heterodera schachtii	**0.002**	0.253	0.149	0.333	0.729	0.771

	Number of seeds per plant			Number of seeds per central plant		
Pieris brassicae	*0.057*	0.389	0.582	**0.022**	0.317	0.446
Athalia rosae	0.945	0.807	0.612	0.825	0.654	0.964
Brevicoryne brassicae	**<0.001**	**<0.001**	0.980	*0.057*	0.137	0.890
Lipaphis erysimi	0.168	0.998	0.164	**0.016**	**0.006**	0.915
Delia radicum	0.897	0.879	0.623	0.990	0.460	0.325
Heterodera schachtii	0.981	0.997	0.994	0.896	0.965	0.763
	Number of seeds per side-plant					
Pieris brassicae	0.400	0.684	0.883			
Athalia rosae	0.973	0.569	0.423			
Brevicoryne brassicae	**<0.001**	**<0.001**	0.873			
Lipaphis erysimi	0.965	0.152	*0.089*			
Delia radicum	0.798	0.989	0.880			
Heterodera schachtii	0.999	0.982	0.969			

Table S3. Output of generalized linear (mixed) models showing the effects of different fixed (herbivore species and plant ontogenetic stage) factors on flower abundance, attraction and visitation by pollinators, and abundance of pollen beetle adults for different time-points. All random factors were initially included in the model, and factors which explained less than 3 percent variation or with a *P* - value above 0.05 were excluded from the model. Bold values indicate results where $P \leq 0.05$. Italic values indicate results where $P \leq 0.1$.

		Herbivore treatment (T)			Plant ontogenetic stage (O)			T*O			Time			Day			Day*T / Plot (Inflor. + PB)			Plant position			Observer		
One week after plants started to produce buds		df	χ^2	*P*	df	χ^2	*P*	df	χ^2	*P*	df	χ^2	*P*	df	χ^2	*P*	df	χ^2	*P*	df	χ^2	*P*	df	χ^2	*P*
Inflorescences	Number	5	25.11	**<0.001**	1	8.36	**0.004**	5	28.54	**<0.001**	-	-	-	-	-	-	1	95.62	**<0.001**	-	-	-			
Pollen beetle adults	Number of visitors	5	11.61	**0.041**	1	4.75	**0.029**	5	25.04	**<0.001**	-	-	-	1	75.30	**<0.001**	1	38.68	**<0.001**	-	-	-			
One week after the start of flowering																									
Inflorescences	Number	5	1.66	0.894	2	0.05	0.975	10	2.32	0.993	-	-	-	-	-	-	1	35.19	**<0.001**	-	-	-	-	-	-
All pollinators	Number of visitors	5	6.21	0.286	2	1.04	0.594	10	41.75	**<0.001**	-	-	-	1	11.64	**<0.001**	1	62.69	**<0.001**	-	-	-	-	-	-
	Flowers visited in total	5	8.33	0.139	1	2.30	0.317	10	13.00	0.224	-	-	-	1	5.41	**0.020**	1	0	1	-	-	-	1	19.40	**<0.001**
Honeybees	Number of visitors	5	7.31	0.199	2	1.66	0.437	10	32.90	**<0.001**	-	-	-	1	8.35	**0.004**	1	55.44	**<0.001**	-	-	-	-	-	-
	Flowers visited in total	5	9.74	*0.083*	2	2.52	0.283	10	7.05	0.721	-	-	-	1	1.94	0.167	1	0	1	-	-	-	1	15.08	**<0.001**
	Flowers visited per visit	5	3.42	0.635	2	4.87	*0.087*	10	6.21	0.797	-	-	-	1	0.19	0.664	-	-	-	-	-	-	-	-	-
	Time spend per plant	5	0.441	0.994	2	2.06	0.356	10	8.59	0.572	1	0	0.989	-	-	-	1	9.03	**0.003**	-	-	-	1	0.38	0.536
	Time spend per flower	5	0.463	0.993	2	4.95	*0.084*	10	17.90	*0.058*	-	-	-	1	3.77	*0.052*	1	10.42	**0.001**	-	-	-	1	17.37	**<0.001**
Bumblebees	Number of visitors	5	5.20	0.392	2	2.22	0.329	9	2.59	0.978	-	-	-	-	-	-	-	-	-	-	-	-	-	-	-
Syrphid flies	Number of visitors	5	4.36	0.499	2	6.50	**0.039**	10	9.81	0.457	-	-	-	1	49.07	**<0.001**	1	3.25	*0.071*	-	-	-	-	-	-
	Flowers visited in total	5	6.46	0.264	2	2.84	0.242	10	17.03	*0.074*	-	-	-	1	0.96	0.328	1	2.56	0.110	-	-	-	1	0	1
	Flowers visited per visit	5	15.40	**0.009**	2	6.91	**0.032**	10	14.82	0.139	-	-	-	1	0	1	-	-	-	-	-	-	1	0	1
	Time spend per plant	5	10.44	*0.064*	2	0.80	0.670	10	10.64	0.386	1	0	1	1	6.90	**0.009**	1	0	1	-	-	-	1	0	1
	Time spend per flower	5	9.21	0.101	2	12.64	**0.002**	10	4.90	0.898	-	-	-	1	0.02	0.899	-	-	-	-	-	-	-	-	-

Pollen beetle adults	Number of visitors	5	5.19	0.394	2	7.36	**0.025**	10	20.37	**0.026**	-	-	-	1	13.87	**<0.001**	1	9.61	**0.002**	1	17.38	**<0.001**	-	-	-
Two weeks after the start of flowering																									
Inflorescences	Number	5	4.07	0.540	2	3.07	0.215	10	21.63	**0.017**	-	-	-	-	-	-	1	9.17	**0.002**	-	-	-	-	-	-
All pollinators	Number of visitors	5	12.09	**0.034**	2	5.56	*0.062*	10	18.72	**0.044**	-	-	-	1	25.59	**<0.001**	1	0	1	-	-	-	-	-	-
	Flowers visited in total	5	7.06	0.216	2	7.16	**0.028**	10	1.81	0.998	1	0	1	1	0.829	0.363	1	0	1	-	-	-	1	5.50	**0.019**
Honeybees	Number of visitors	5	11.52	**0.042**	2	6.47	**0.039**	10	17.33	*0.067*	-	-	-	1	19.61	**<0.001**	1	0	1	-	-	-	-	-	-
	Flowers visited in total	5	8.44	0.134	2	9.15	**0.010**	10	4.73	0.908	1	0	0.999	-	-	-	1	0	1	-	-	-	1	3.50	*0.061*
	Flowers visited per visit	5	3.08	0.687	2	8.40	**0.015**	10	15.35	0.120	-	-	-	1	1.42	0.233	1	82.65	**<0.001**	-	-	-	-	-	-
	Time spend per plant	5	2.61	0.760	2	0.04	0.981	10	6.57	0.765	1	0	**1**	-	-	-	-	-	-	-	-	-	-	-	-
	Time spend per flower	5	6.99	0.221	2	11.05	**0.004**	10	10.45	0.402	-	-	-	-	-	-	-	-	-	-	-	-	1	0.31	0.579
Bumblebees	Number of visitors*	-	-	-	2	4.53	0.104	-	-	-	-	-	-	-	-	-	-	-	-	-	-	-	-	-	-
Syrphid flies	Number of visitors	5	17.81	**0.003**	2	0.800	0.670	10	6.66	0.758	-	-	-	1	85.85	**<0.001**	-	-	-	-	-	-	-	-	-
	Flowers visited in total*	-	-	-	3	1.55	0.671	-	-	-	-	-	-	1	0	1	-	-	-	-	-	-	-	-	-
	Flowers visited per visit*	-	-	-	3	0.37	0.946	-	-	-	1	0.03	0.860	1	0.31	0.577	-	-	-	-	-	-	1	0	1
	Time spend per plant*	-	-	-	3	6.10	0.107	-	-	-	-	-	-	-	-	-	-	-	-	-	-	-	1	0	1
	Time spend per flower*	-	-	-	3	4.38	0.223	-	-	-	1	0	1	-	-	-	-	-	-	-	-	-	1	0	1
Pollen beetle adults	Number of visitors	5	7.30	0.200	2	0.10	0.953	10	17.92	*0.056*	-	-	-	1	59.23	**<0.001**	1	16.74	**<0.001**	1	15.46	**<0.001**	-	-	-

*At two weeks after plants had started flowering, we collected a limited number of observations for bumblebees and syrphid flies. Therefore, we could not analyse the effects of plant exposure to different herbivore species on their visitation behaviour. Plots were grouped based on plant ontogenetic stage for these analyses.

Table S4. Output of generalized linear (mixed) models showing the effects of different fixed (herbivore species, time-point, and herbivore functional group) factors on flower abundance, attraction and visitation by pollinators, and abundance of pollen beetle adults, for herbivore exposure in different plant ontogenetic stages. All random factors were initially included in the model, and factors which explained less than 3 percent variation or with a *P* - value above 0.05 were excluded from the model. Bold values indicate results where $P \leq 0.05$. Italic values indicate results where $P \leq 0.1$.

		Herbivore treatment (T)			Time point (TP)			T*TP			Herbivore functional group (HFG)			Day			Day*T / Plot (Inflor. + PB)			Time / Plant position (Inflor. + PB)			Observer		
Vegetative stage		df	χ^2	*P*	df	χ^2	*P*	df	χ^2	*P*	df	χ^2	*P*	df	χ^2	*P*	df	χ^2	*P*	df	χ^2	*P*	df	χ^2	*P*
Inflorescences	Number	3	23.19	**<0.001**	2	728.81	**<0.001**	12	139.52	**<0.001**	3	7.04	*0.070*	-	-	-	1	22.72	**<0.001**	-	-	-	-	-	-
All pollinators	Number of visitors	3	8.21	**0.042**	1	4.73	**0.030**	6	30.65	**<0.001**	3	1.27	0.736	1	7.75	**0.005**	1	88.34	**<0.001**	-	-	-	-	-	-
	Flowers visited in total	3	1.63	0.653	1	1.75	0.186	6	4.58	0.598	3	0.80	0.850	1	1.12	0.290	1	0	1	-	-	-	1	9.79	**0.002**
Honeybees	Number of visitors	3	7.72	*0.052*	1	3.35	*0.067*	6	24.11	**<0.001**	3	0.733	0.865	1	4.40	**0.036**	1	78.02	**<0.001**	-	-	-	-	-	-
	Flowers visited in total	3	2.00	0.572	1	4.31	**0.038**	6	5.78	0.449	3	1.44	0.697	1	0.24	0.623	1	0	1	-	-	-	1	11.83	**<0.001**
	Flowers visited per visit	3	2.33	0.507	1	8.60	**0.003**	6	23.16	**<0.001**	3	1.05	0.789	1	2.23	0.135	1	256.79	**<0.001**	-	-	-	-	-	-
	Time spend per plant	3	2.31	0.510	1	0.10	0.752	6	2.29	0.891	3	1.83	0.609	1	0.72	0.396	1	0	1	-	-	-	-	-	-
	Time spend per flower	3	1.72	0.632	1	0.115	0.735	6	12.65	**0.049**	3	2.12	0.548	1	0	1	-	-	-	-	-	-	1	4.36	**0.037**
Bumblebees	Number of visitors*	3	0.314	0.957	-	-	-	-	-	-	3	0.400	0.940	-	-	-	-	-	-	-	-	-	-	-	-
Syrphid flies	Number of visitors	3	8.12	**0.044**	1	8.38	**0.004**	3	3.63	0.726	3	2.07	0.558	1	32.13	**<0.001**	1	1.96	0.161	-	-	-	-	-	-
	Flowers visited in total*	3	1.34	0.721	-	-	-	-	-	-	3	6.99	*0.072*	1	0.18	0.672	1	345.23	**<0.001**	-	-	-	1	7.91	**0.005**
	Flowers visited per visit*	3	16.49	**<0.001**	-	-	-	-	-	-	3	32.32	**<0.001**	1	145.51	**<0.001**	1	2.60	0.107	-	-	-	1	17.59	**<0.001**
	Time spend per plant*	3	9.56	**0.023**	-	-	-	-	-	-	3	5.30	0.151	1	12.60	**<0.001**	1	0	1	-	-	-	1	1.78	0.182
	Time spend per flower*	3	2.26	0.521	-	-	-	-	-	-	3	11.20	**0.011**	-	-	-	-	-	-	1	1.30	0.255	-	-	-
Pollen beetle adults	Number of visitors	3	3.19	0.364	2	35.33	**<0.001**	12	64.07	**<0.001**	3	3.98	0.264	1	208.89	**<0.001**	1	23.61	**<0.001**	1	16.56	**<0.001**	-	-	-

Bud stage																									
Inflorescences	Number	3	0.59	0.899	2	592.0	**<0.001**	12	29.64	**0.003**	3	4.06	0.255	-	-	-	1	15.08	**<0.001**	-	-	-	-	-	-
All pollinators	Number of visitors	3	4.44	0.218	1	1.84	0.175	6	16.91	**0.010**	3	0.10	0.992	1	14.96	**<0.001**	1	69.04	**<0.001**	1	13.75	**<0.001**	1	27.62	**<0.001**
	Flowers visited in total	3	7.51	*0.057*	1	1.48	0.225	6	10.19	0.117	3	4.51	0.212	1	0.02	0.883	1	0	0.998	-	-	-	1	12.50	**<0.001**
Honeybees	Number of visitors	3	4.50	0.212	1	36.77	**<0.001**	6	14.80	**0.022**	3	0.09	0.993	1	11.56	**<0.001**	1	47.92	**<0.001**	-	-	-	1	30.28	**<0.001**
	Flowers visited in total	3	3.36	0.340	1	4.31	**0.038**	6	11.43	*0.076*	3	3.32	0.345	1	0.01	0.936	1	0	0.995	-	-	-	1	15.93	**<0.001**
	Flowers visited per visit	3	1.62	0.655	1	2.03	0.154	6	6.83	0.337	3	0.735	0.865	1	5.66	**0.017**	1	56.46	**<0.001**	-	-	-	-	-	-
	Time spend per plant	3	3.13	0.372	1	0.08	0.772	6	6.65	0.355	3	1.11	0.774	1	13.66	**<0.001**	1	0.367	0.545	1	2.59	0.108	-	-	-
	Time spend per flower	6	12.92	**0.005**	1	1.16	0.282	6	6.75	0.345	3	8.40	**0.038**	1	0.73	0.394	-	-	-	-	-	-	1	3.45	*0.063*
Bumblebees	Number of visitors*	3	2.58	0.461	-	-	-	-	-	-	3	3.74	0.291	-	-	-	-	-	-	-	-	-	-	-	-
Syrphid flies	Number of visitors	3	1.81	0.613	1	6.46	**0.011**	6	14.10	**0.029**	3	2.97	0.397	1	12.55	**<0.001**	1	4.86	**0.027**	-	-	-	-	-	-
	Flowers visited in total*	3	4.34	0.227	-	-	-	-	-	-	3	1.81	0.614	1	4.31	**0.038**	1	106.91	**<0.001**	-	-	-	1	11.98	**<0.001**
	Flowers visited per visit*	3	6.00	0.112	-	-	-	-	-	-	3	73.34	**<0.001**	1	61.42	**<0.001**	-	-	-	-	-	-	1	31.62	**<0.001**
	Time spend per plant*	3	1.51	0.680	-	-	-	-	-	-	3	7.92	**0.048**	1	4.29	**0.038**	-	-	-	-	-	-	1	0	1
	Time spend per flower*	3	5.16	0.161	-	-	-	-	-	-	3	0.16	0.984	1	0.28	0.599	1	1.00	0.318	-	-	-	-	-	-
Pollen beetle adults	Number of visitors	3	5.45	0.142	2	69.10	**<0.001**	12	32.39	**0.001**	3	8.16	**0.043**	1	177.58	**<0.001**	1	11.93	**<0.001**	1	13.33	**<0.001**	-	-	-
Flowering stage																									
Inflorescences	Number	3	0.41	0.939	1	11.78	**<0.001**	6	6.04	0.418	3	1.40	0.705	-	-	-	1	100.72	**<0.001**	1	82.23	**<0.001**	-	-	-
All pollinators	Number of visitors	3	6.51	*0.089*	1	18.85	**<0.001**	6	21.75	**0.001**	3	1.29	0.730	1	13.54	**<0.001**	1	25.21	**<0.001**	-	-	-	-	-	-
	Flowers visited in total	3	0.48	0.924	1	6.18	**0.013**	6	3.07	0.800	3	7.47	*0.058*	1	0.559	0.455	1	0	1	-	-	-	1	19.60	**<0.001**
Honeybees	Number of visitors	3	6.54	*0.088*	1	14.51	**<0.001**	6	16.48	**0.011**	3	1.28	0.735	1	10.90	**<0.001**	1	20.85	**<0.001**	-	-	-	-	-	-
	Flowers visited in total	3	0.61	0.893	1	1.81	0.178	6	3.19	0.785	3	16.09	**0.001**	-	-	-	1	0	1	-	-	-	-	-	-

	Flowers visited per visit	3	2.14	0.544	1	0.04	0.835	6	8.77	0.187	3	3.76	0.288	-	-	-	1	168.35	**<0.001**	-	-	-	-	-	-
	Time spend per plant	3	4.14	0.247	1	1.91	0.167	6	2.81	0.832	3	3.03	0.387	-	-	-	-	-	-	1	0.317	0.574	1	1.20	0.274
	Time spend per flower	3	0.122	0.989	1	0.269	0.604	6	15.56	**0.016**	3	1.25	0.740	1	6.19	**0.013**	-	-	-	-	-	-	1	37.28	**<0.001**
Bumblebees	Number of visitors*	2	0.01	0.997	-	-	-	-	-	-	3	0.623	0.891	-	-	-	-	-	-	-	-	-	-	-	-
Syrphid flies	Number of visitors	3	2.17	0.538	1	3.21	*0.073*	6	5.14	0.527	3	5.79	0.122	1	51.78	**<0.001**	-	-	-	-	-	-	-	-	-
	Flowers visited in total*	3	3.24	0.356	-	-	-	-	-	-	3	3.49	0.322	1	3.54	*0.060*	1	422.35	**<0.001**	-	-	-	-	-	-
	Flowers visited per visit*	3	13.36	**0.004**	-	-	-	-	-	-	3	15.52	**0.001**	1	283.97	**<0.001**	-	-	-	-	-	-	1	4.71	**0.030**
	Time spend per plant*	3	13.90	**0.003**	-	-	-	-	-	-	3	3.50	0.320	1	5.34	**0.021**	-	-	-	1	0	1	-	-	-
	Time spend per flower*	3	4.70	0.195	-	-	-	-	-	-	3	6.61	*0.085*	1	3.10	*0.078*	1	0	1	1	0	1	-	-	-
Pollen beetle adults	Number of visitors	3	2.42	0.490	1	14.73	**<0.001**	6	6.19	0.402	3	4.70	0.195	1	97.06	**<0.001**	1	13.10	**<0.001**	1	22.10	**<0.001**	-	-	-

*At two weeks after plants had started flowering, we collected a limited number of observations for bumblebees and syrphid flies. Therefore, we limited these analyses to only include data for observations at one week after plants had started flowering.

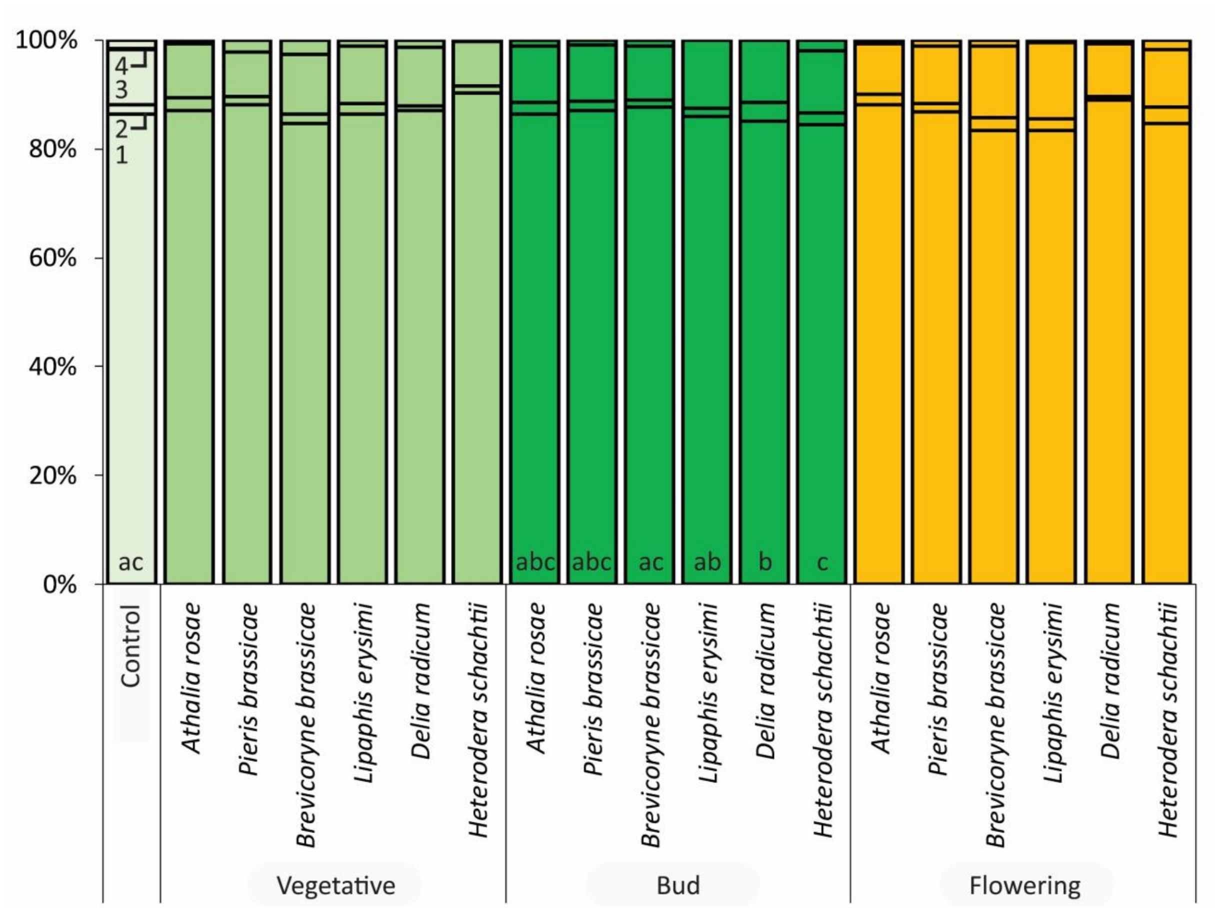

Fig. S2 Composition of pollinator communities of uninfested plots (control) of *Brassica nigra* plants and plots infested by herbivores at different plant ontogenetic stages. Communities consist of honeybees (1), bumblebees (2), syrphid flies (3), and solitary bees (4). Letters at the bottom of the bars indicate marginally insignificant differences for $0.05 < P < 0.1$ between herbivore species within a plant ontogenetic stage based on pairwise chi-square tests.

Table S5. Standardized residuals for the number of visitors of each pollinator group (honeybees, bumblebees, syrphid flies, and solitary bees) for uninfested *Brassica nigra* plants or plants infested with herbivores at different plant ontogenetic stages. Numbers displayed in bold exceed the ± 2 criteria.

	Uninfested (control)					
Honeybees	0.05					
Bumblebees	-0.12					
Syrphid flies	-0.48					
Solitary bees	1.36					
Vegetative	*Athalia rosae*	*Pieris brassicae*	*Brevicoryne brassicae*	*Lipaphis erysimi*	*Delia radicum*	*Heterodera schachtii*
Honeybees	0.55	1.05	-1.10	0.04	0.50	**2.02**
Bumblebees	0.72	-0.76	-0.13	0.01	-1.74	-0.74
Syrphid flies	-0.56	-1.47	0.22	-0.06	-0.02	-1.44
Solitary bees	-1.06	1.83	**3.09**	0.03	0.64	-1.38
Bud						
Honeybees	-0.02	0.39	0.94	-0.32	-0.88	-1.38
Bumblebees	0.52	0.04	-1.08	-0.56	**2.78**	0.69
Syrphid flies	-0.19	-0.23	-0.52	1.43	0.60	0.53
Solitary bees	-0.07	-0.66	-0.16	**-2.38**	**-2.43**	**2.04**
Flowering						
Honeybees	1.23	0.27	-1.99	-1.73	1.63	-1.05
Bumblebees	-0.11	-0.44	1.00	0.16	-1.97	1.75
Syrphid flies	-1.05	-0.14	1.71	**2.30**	-0.59	-0.07
Solitary bees	-0.76	0.10	0.17	-1.27	-1.03	1.35

Delia radicum	Vegetative	Bud	Flowering
Honeybees	0.08	-1.54	1.48
Bumblebees	-1.79	**3.88**	**-2.04**
Syrphid flies	-0.02	0.71	-0.69
Solitary bees	**2.45**	**-2.29**	-0.25
Heterodera schachtii			
Honeybees	**2.52**	-1.32	-0.89
Bumblebees	-1.31	-0.02	1.21
Syrphid flies	-1.42	1.03	0.21
Solitary bees	**-2.01**	1.26	0.54

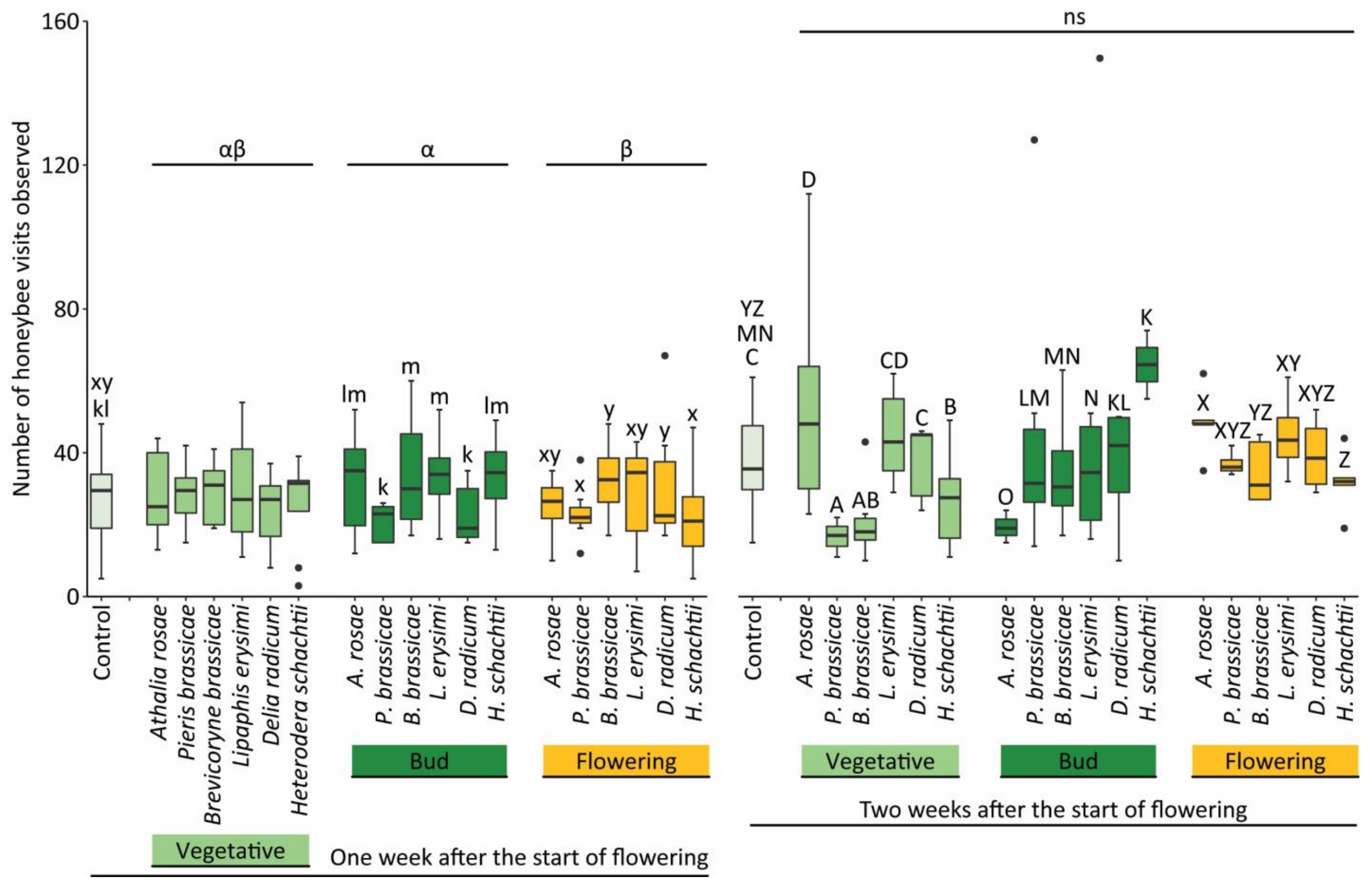

Fig. S3 Number of honeybee visits observed on flowers of uninfested plots (control) of *Brassica nigra* plants and on flowers of plots infested by herbivores at different plant ontogenetic stages. Boxplots show median (line), 1st and 3rd quartiles, minimum and maximum. Outliers (1.5 times the interquartile range below the 1st or above the 3rd quartile) are represented by circles. Observations lasted for 10 min and were made at two time points: between 7 - 9 days and between 14 - 16 days after plots had started flowering. For 7 - 9 days after plots had started flowering, the number of replicates per herbivore treatment varied between 7 and 9, and 16 for the control treatment. For 14 - 16 days after plots had started flowering, the number of replicates per herbivore treatment varied between 2 and 6, and was 10 for the control treatment. Letter groups (a - d, k - n, x - z) above bars indicate significant differences at ($P \leq 0.05$) between herbivore species within a plant ontogenetic stage based on Tukey's *post hoc* tests, and small or capital letters were used for different time-points. Greek letters above lines indicate significant differences at ($P \leq 0.05$) between plant ontogenetic stages based on Tukey's *post hoc* tests, whereas *ns* indicates no differences.

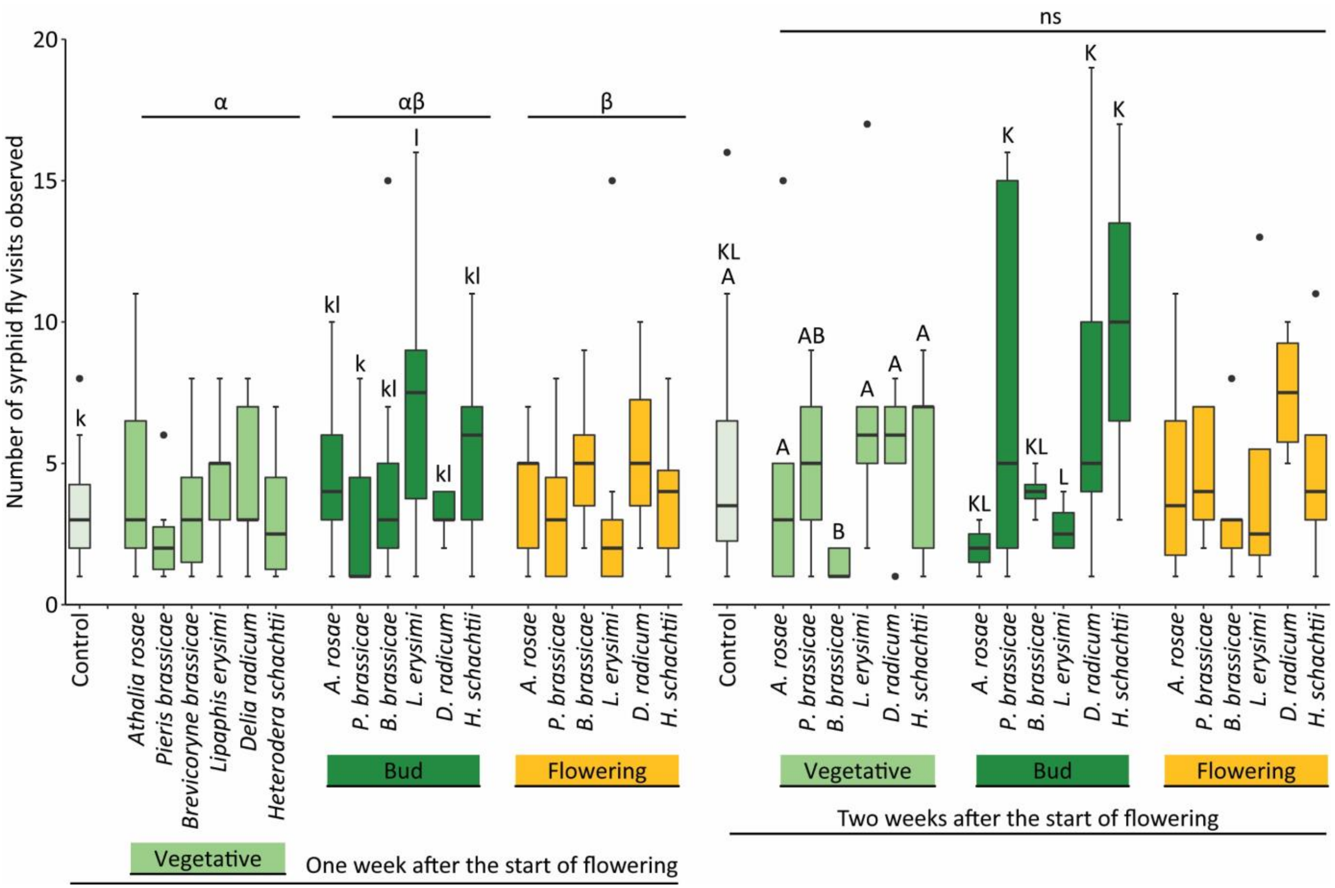

Fig. S4 Number of syrphid fly visits observed on flowers of uninfested plots (control) of *Brassica nigra* plants and on flowers of plots infested by herbivores at different plant ontogenetic stages. Boxplots show median (line), 1st and 3rd quartiles, minimum and maximum. Outliers are represented by circles (1.5 times the interquartile range below the 1st or above the 3rd quartile). Observations lasted for 10 min and were made at two time points: 7 - 9 and 14 - 16 days after plots had started flowering. For 7 - 9 days after plots had started flowering, the number of replicates per herbivore treatment varied between 5 and 8, and 12 for the control treatment. For 14 - 16 days after plots had started flowering, the number of replicates per herbivore treatment varied between 2 and 5, and was 10 for the control treatment. Letter groups (a - b, k - l) above bars indicate significant differences at ($P \leq 0.05$) between herbivore species within a plant ontogenetic stage based on Tukey's *post hoc* tests, and small or capital letters were used for different time-points. Greek letters above lines indicate significant differences at ($P \leq 0.05$) between plant ontogenetic stages based on Tukey's *post hoc* tests, whereas *ns* indicates no differences.

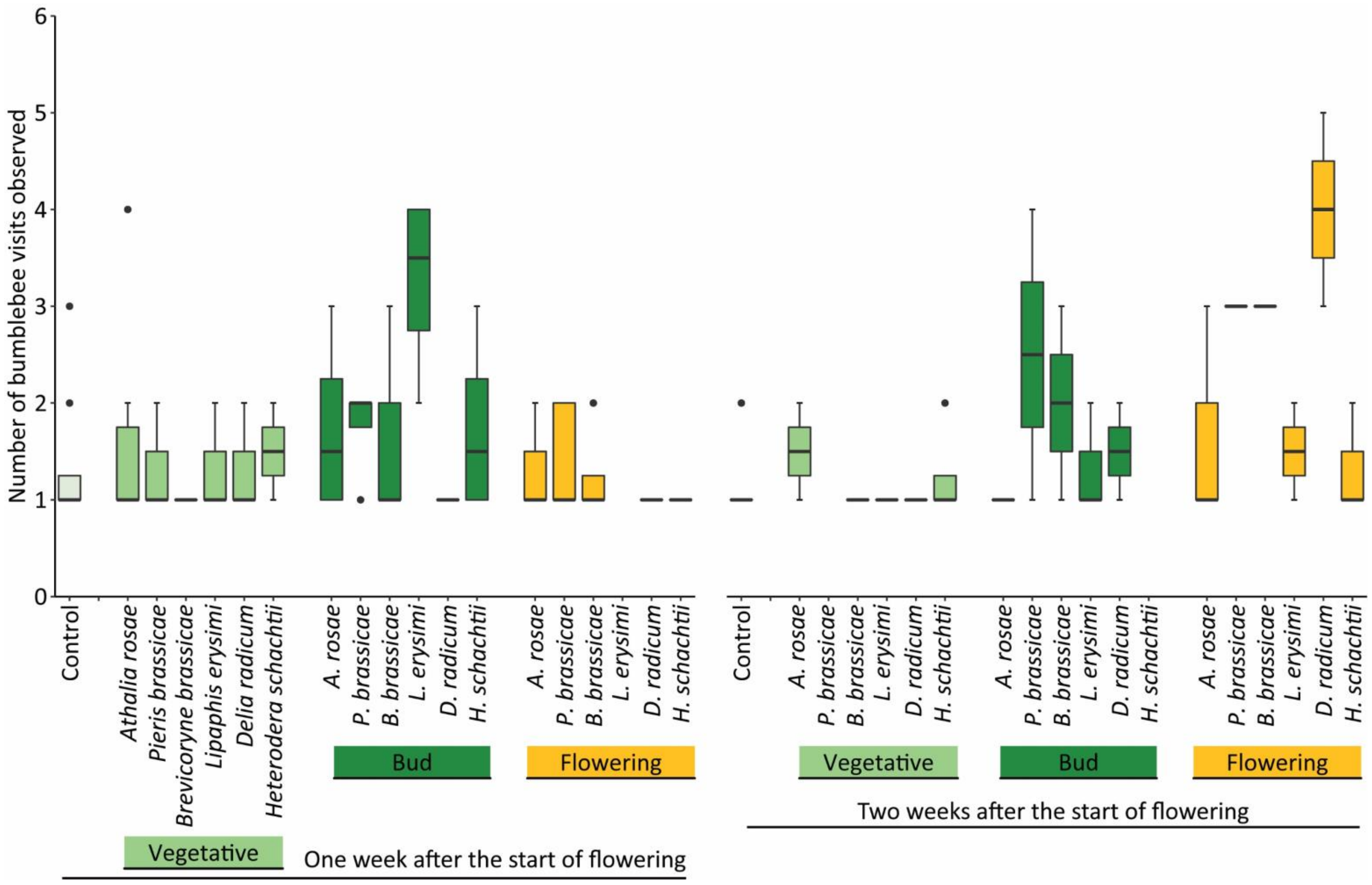

Fig. S5 Number of bumblebee visits observed on flowers of uninfested plots (control) of *Brassica nigra* plants and on flowers of plots infested by herbivores at different plant ontogenetic stages. Boxplots show median (line), 1st and 3rd quartiles, minimum and maximum. Outliers (1.5 times the interquartile range below the 1st or above the 3rd quartile) are represented by circles. Observations lasted for 10 min and were made at two time points: between 7 - 9 days and between 14 - 16 days after plots had started flowering. For 7 - 9 days after plots had started flowering, the number of replicates per herbivore treatment varied between 0 and 6, and was 8 for the control treatment. For 14 - 16 days after plots had started flowering, the number of replicates per herbivore treatment varied between 0 and 4, and was 5 for the control treatment.

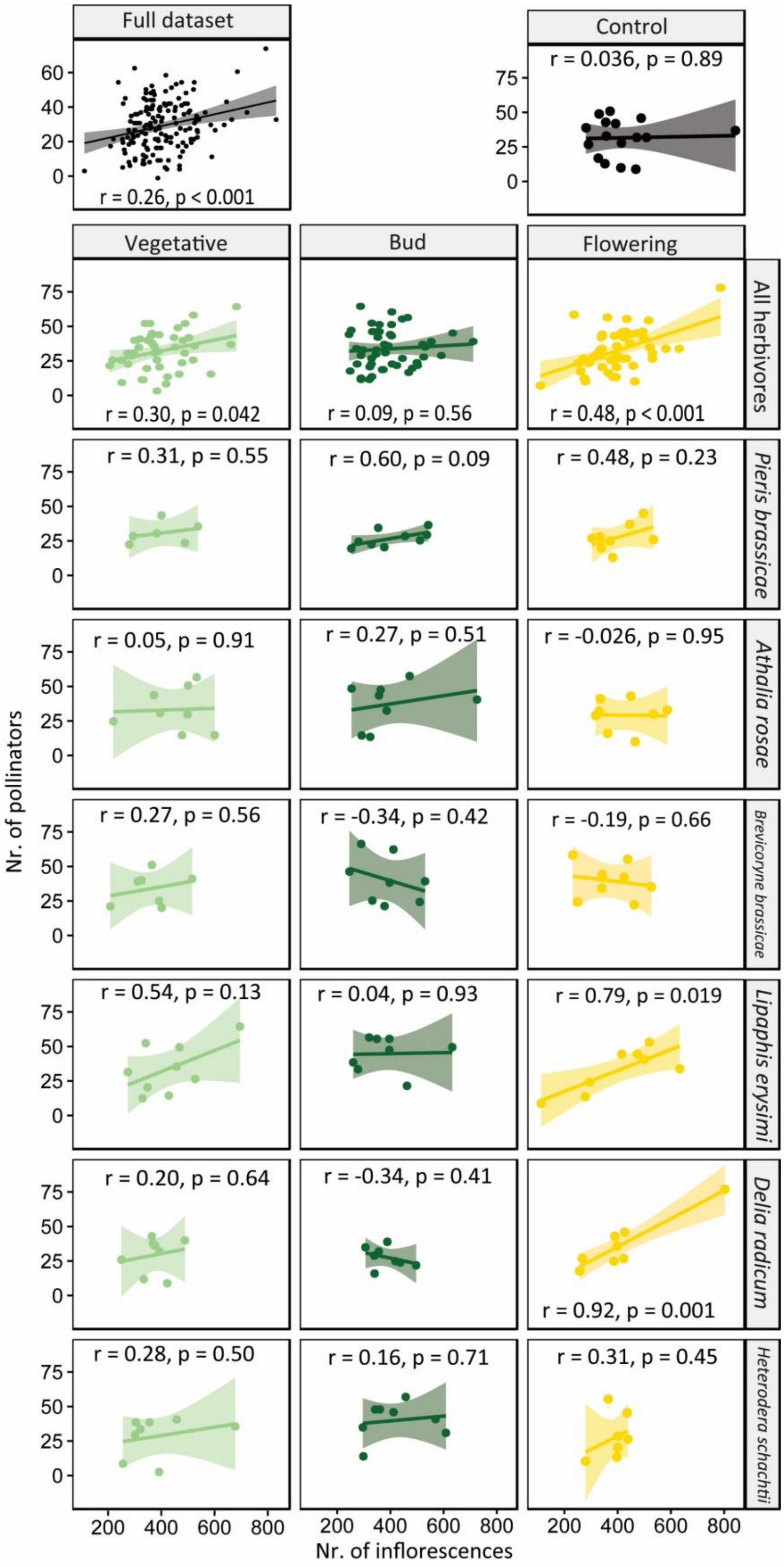

Fig. S6 Number of pollinator visits to plots of *Brassica nigra* plants with different numbers of inflorescences one week after plants in the plots had started flowering. Plots were uninfested (control) or infested with herbivores at different plant ontogenetic stages. Number of inflorescences per plot is the sum of 5 plants. Correlation coefficient r was computed using the Pearson method.

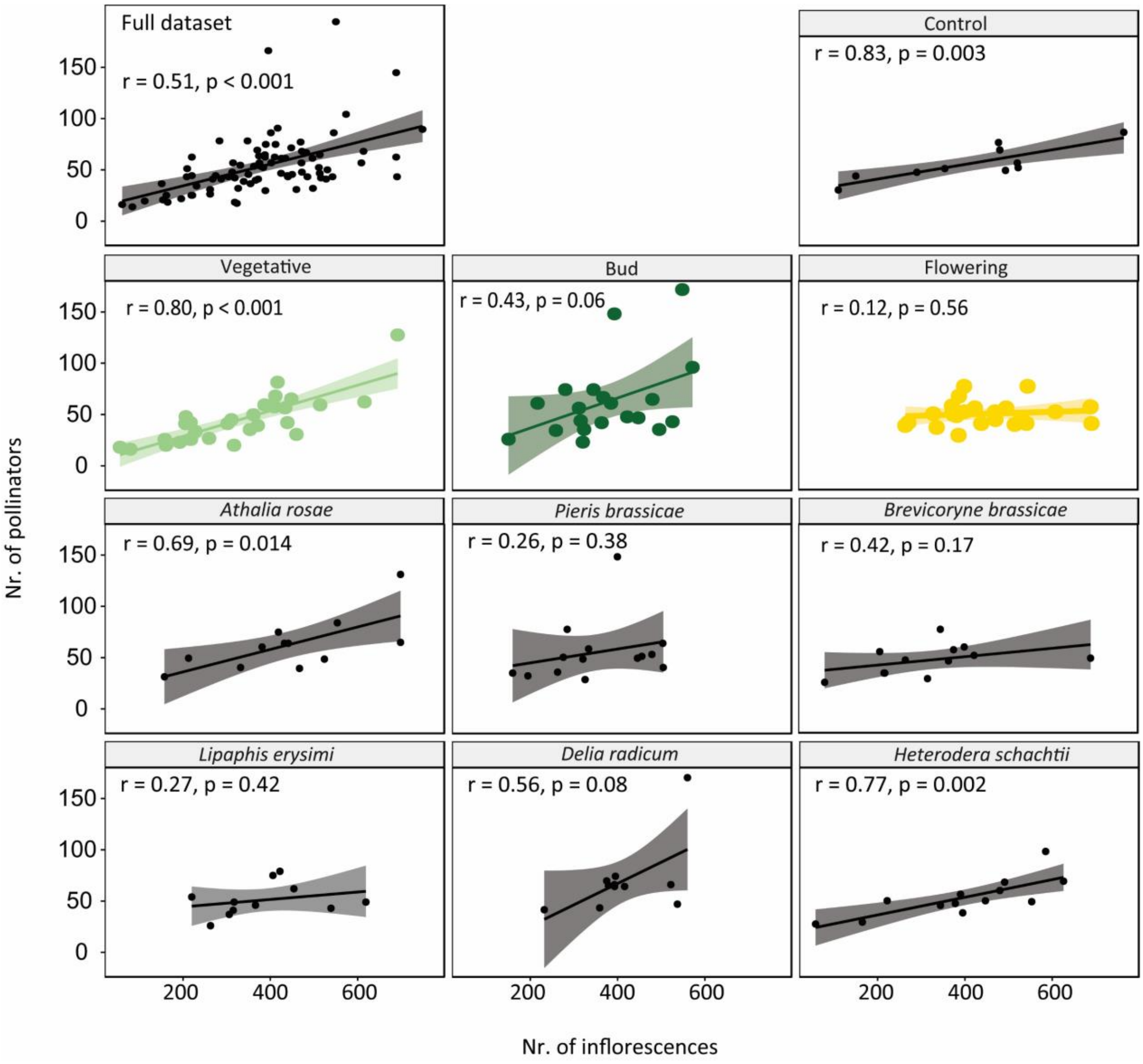

Fig. S7 Number of pollinators visits to plots of *Brassica nigra* plants with different numbers of inflorescences two weeks after plants in the plots had started flowering. Plots were uninfested (control) or infested with herbivores at different plant ontogenetic stages. Number of inflorescences per plot is the sum of 5 plants. Correlation coefficient r was computed using the Pearson method.

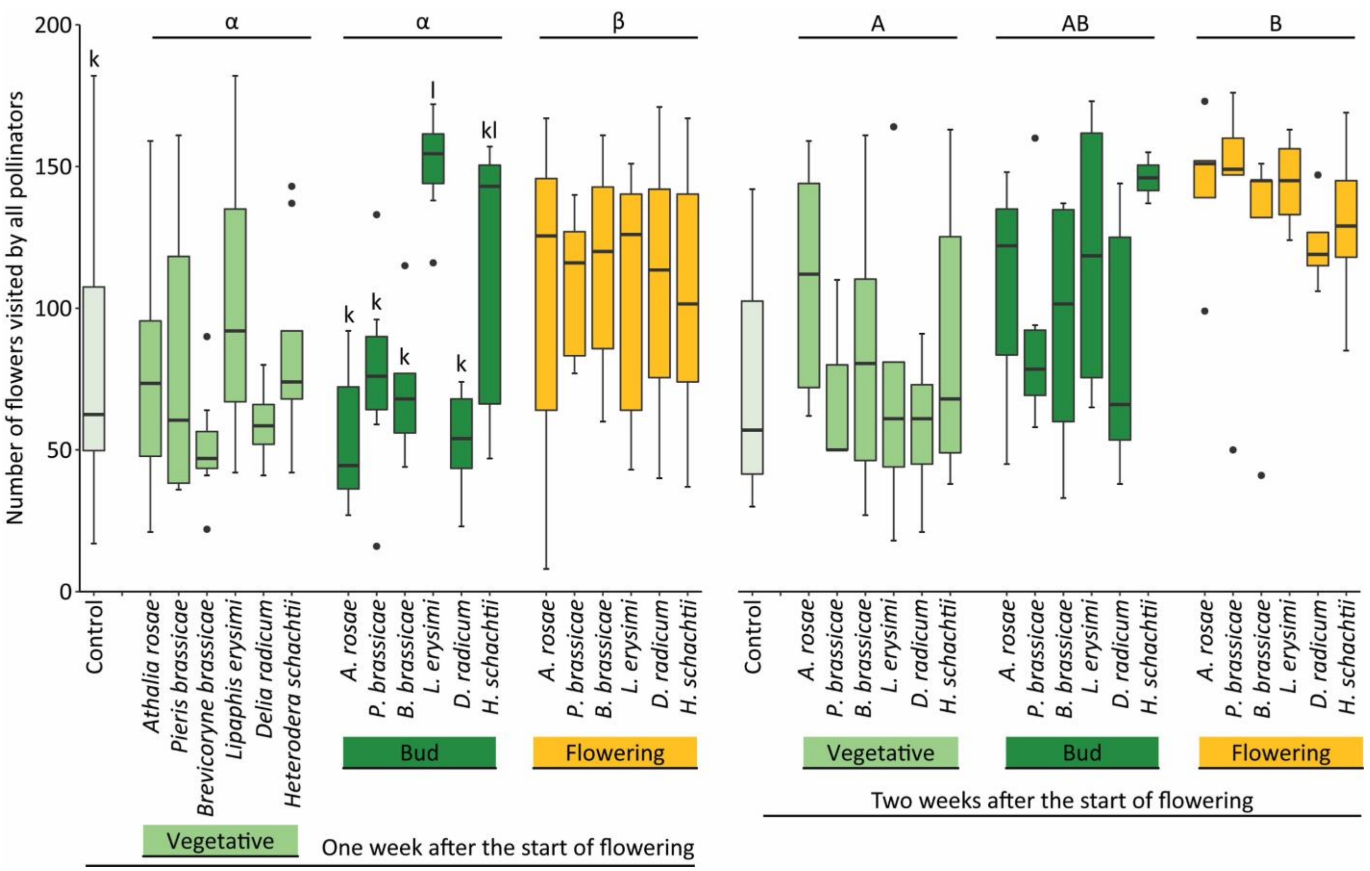

Fig. S8 Number of flowers visited by all pollinators on uninfested plots (control) of *Brassica nigra* plants and on plots infested by herbivores at different plant ontogenetic stages. Boxplots show median (line), 1st and 3rd quartiles, minimum and maximum. Outliers (1.5 times the interquartile range below the 1st or above the 3rd quartile) are represented by circles. Observations lasted for 10 min and were made at two time points: between 7 - 9 days and between 14 - 16 days after plots had started flowering. For 7 - 9 days after plots had started flowering, the number of replicates per herbivore treatment varied between 7 and 9, and was 16 for the control treatment. For 14 - 16 days after plots had started flowering, the number of replicates per herbivore treatment varied between 2 and 6, and was 10 for the control treatment. Letters group (k - l) above bars indicate significant differences at ($P \leq 0.05$) between herbivore species within a plant ontogenetic stage based on Tukey's *post hoc* tests. Greek letters above lines indicate significant differences at ($P \leq 0.05$) between plant ontogenetic stages based on Tukey's *post hoc* tests, and small or capital letters were used for different time-points.

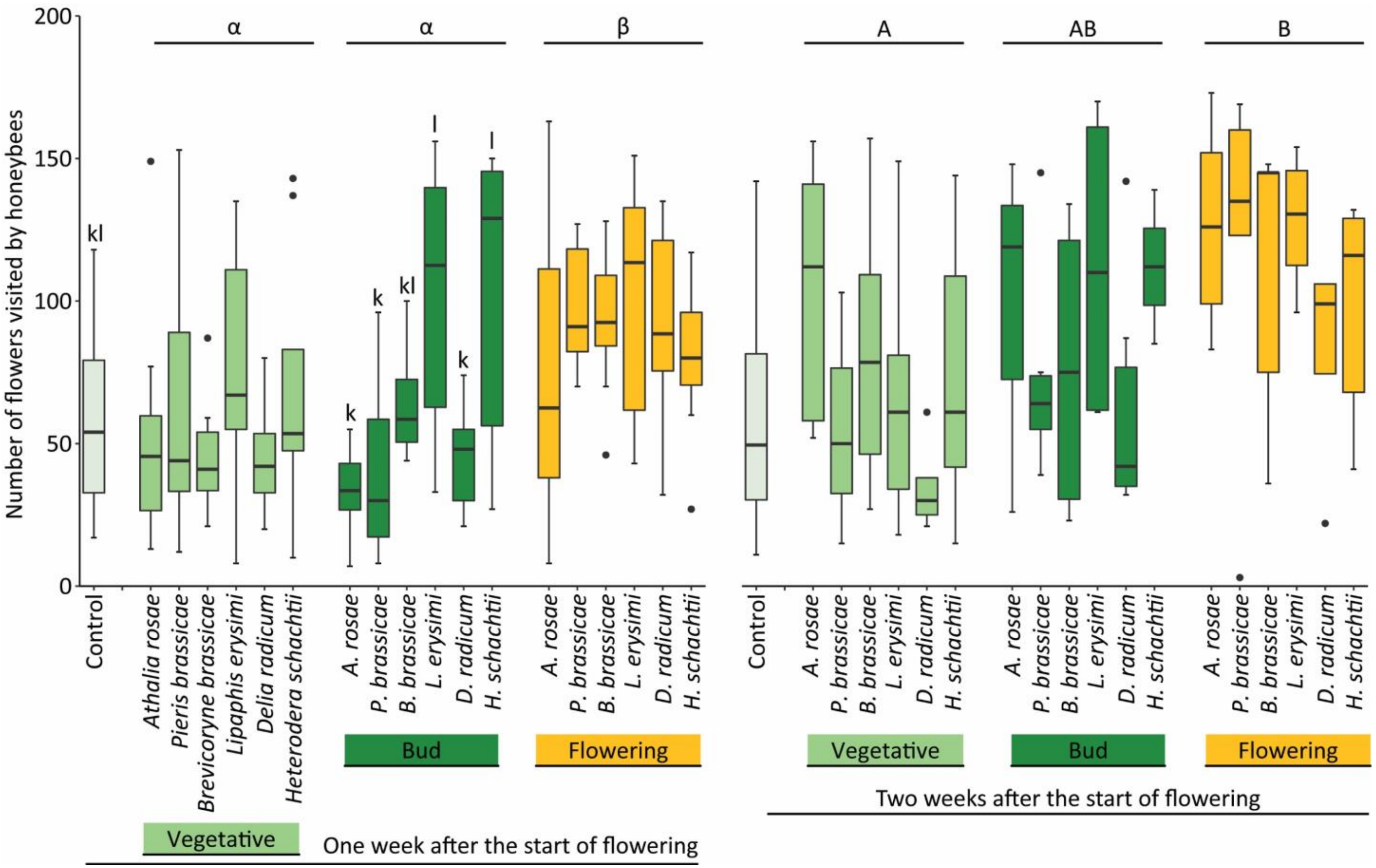

Fig. S9 Number of flowers visited by honeybees on uninfested plots (control) of *Brassica nigra* plants and on plots infested by herbivores at different plant ontogenetic stages. Boxplots show median (line), 1st and 3rd quartiles, minimum and maximum. Outliers (1.5 times the interquartile range below the 1st or above the 3rd quartile) are represented by circles. Observations lasted for 10 min and were made at two time points: between 7 - 9 days and between 14 - 16 days after plots had started flowering. For 7 - 9 days after plots had started flowering, the number of replicates per herbivore treatment varied between 7 and 9, and was 16 for the control treatment. For 14 - 16 days after plots had started flowering, the number of replicates per herbivore treatment varied between 2 and 6, and was 10 for the control treatment. Letters group (k - l) above bars indicate significant differences at ($P \leq 0.05$) between herbivore species within a plant ontogenetic stage based on Tukey's *post hoc* tests. Greek letters above lines indicate significant differences at ($P \leq 0.05$) between plant ontogenetic stages based on Tukey's *post hoc* tests and small or capital letters were used for different time-points.

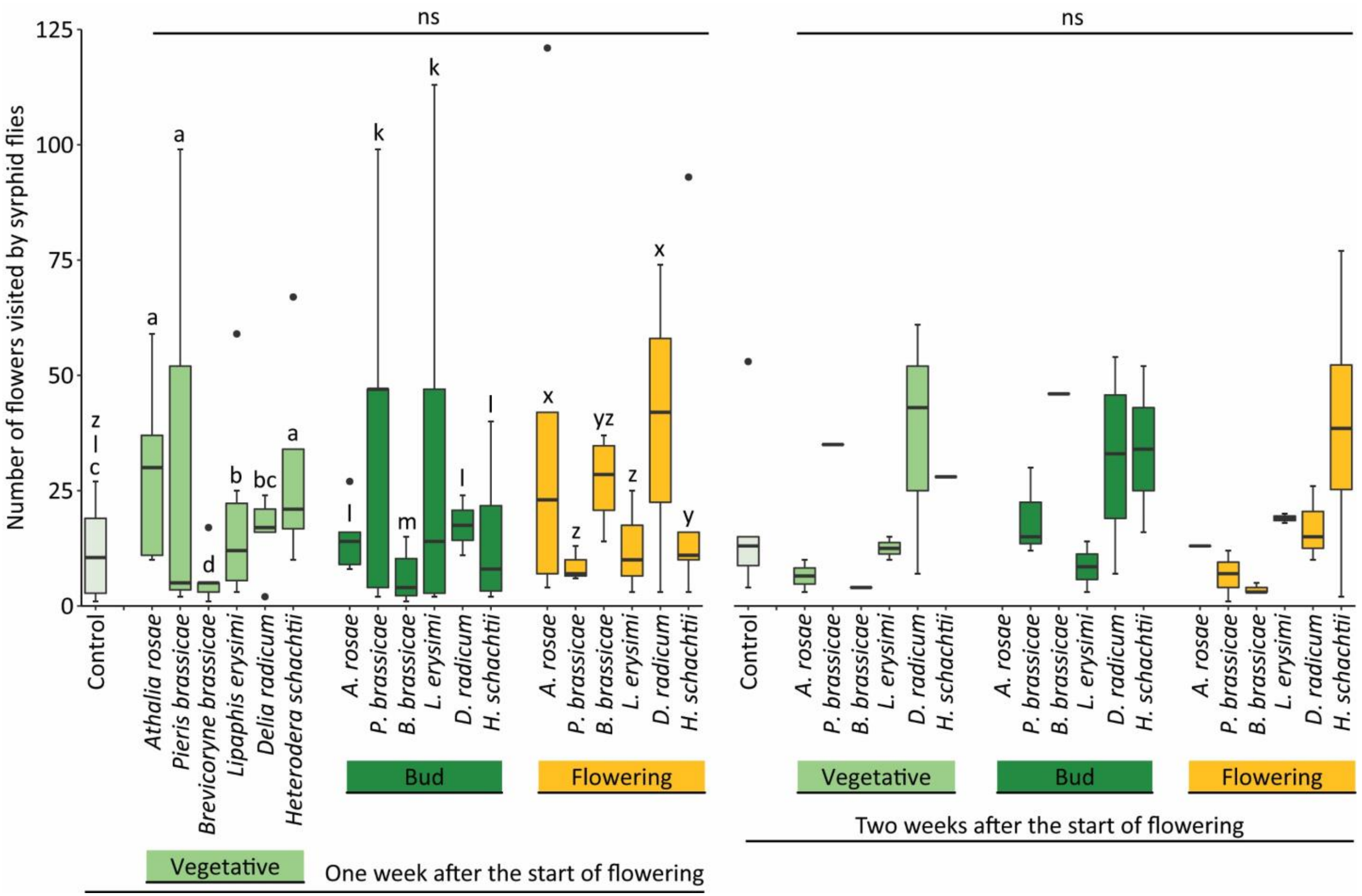

Fig. S10 Number of flowers visited by syrphid flies on uninfested plots (control) of *Brassica nigra* plants and on plots infested by herbivores at different plant ontogenetic stages. Boxplots show median (line), 1st and 3rd quartiles, minimum and maximum. Outliers (1.5 times the interquartile range below the 1st or above the 3rd quartile) are represented by circles. Observations lasted for 10 min and were made at two time points: between 7 – 9 days and between 14 and 16 days after plots had started flowering. For 7 - 9 days after plots had started flowering, the number of replicates per herbivore treatment varied between 2 and 6, and was 8 for the control treatment. For 14 - 16 days after plots had started flowering, the number of replicates per herbivore treatment varied between 0 and 4, and was 6 for the control treatment. Letter groups (a - d, k - l, x - z) above bars indicate significant differences at ($P \leq 0.05$) between herbivore species within a plant ontogenetic stage based on Tukey's *post hoc* tests. *ns* indicates no differences between plant ontogenetic stages based on Tukey's *post hoc* tests.

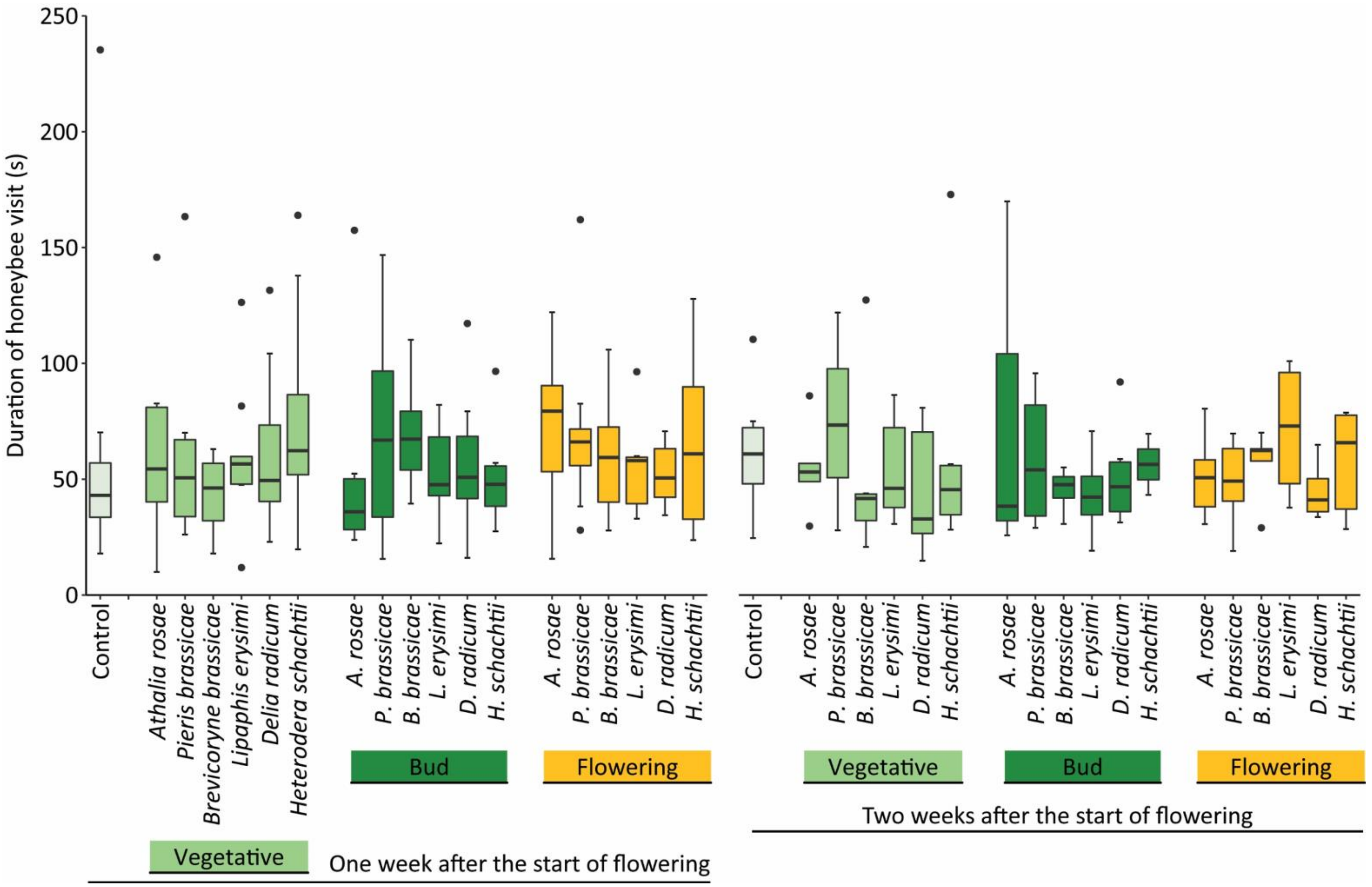

Fig. S11 Duration of honeybee visits to uninfested plots (control) of *Brassica nigra* plants and to plots infested by herbivores at different plant ontogenetic stages. Boxplots show median (line), 1st and 3rd quartiles, minimum and maximum. Outliers (1.5 times the interquartile range below the 1st or above the 3rd quartile) are represented by circles. Observations lasted for 10 min and were made at two time points: between 7 – 9 days and between 14 - 16 days after plots had started flowering. For 7 - 9 days after plots had started flowering, the number of replicates per herbivore treatment varied between 7 and 9, and was 16 for the control treatment. For 14 - 16 days after plots had started flowering, the number of replicates per herbivore treatment varied between 2 and 6, and was 10 for the control treatment.

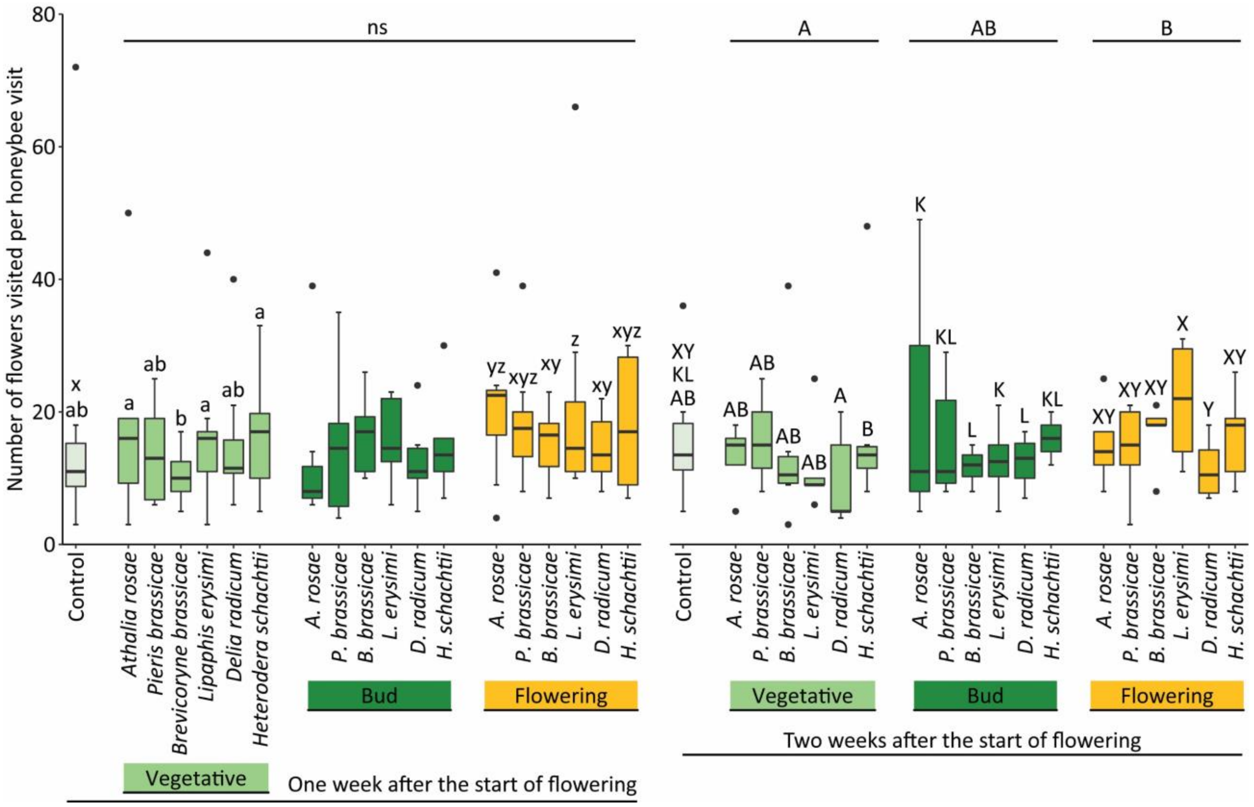

Fig. S12 Number of flowers visited per honeybee visit to uninfested plots (control) of *Brassica nigra* plants and to plots infested by herbivores at different plant ontogenetic stages. Boxplots show median (line), 1st and 3rd quartiles, minimum and maximum. Outliers (1.5 times the interquartile range below the 1st or above the 3rd quartile) are represented by circles. Observations lasted for 10 min and were made at two time points: between 7 – 9 days and between 14 - 16 days after plots had started flowering. For 7 - 9 days after plots had started flowering, the number of replicates per herbivore treatment varied between 7 and 9, and was 16 for the control treatment. For 14 - 16 days after plots had started flowering, the number of replicates per herbivore treatment varied between 2 and 6, and was 10 for the control treatment. Letter groups (a - b, k - l, x - z) above bars indicate significant differences at ($P \leq 0.05$) between herbivore species within a plant ontogenetic stage based on Tukey's *post hoc* tests, and small or capital letters were used for different time-points. Greek letters above lines indicate significant differences at ($P \leq 0.05$) between plant ontogenetic stages based on Tukey's *post hoc* tests, whereas *ns* indicates no differences.

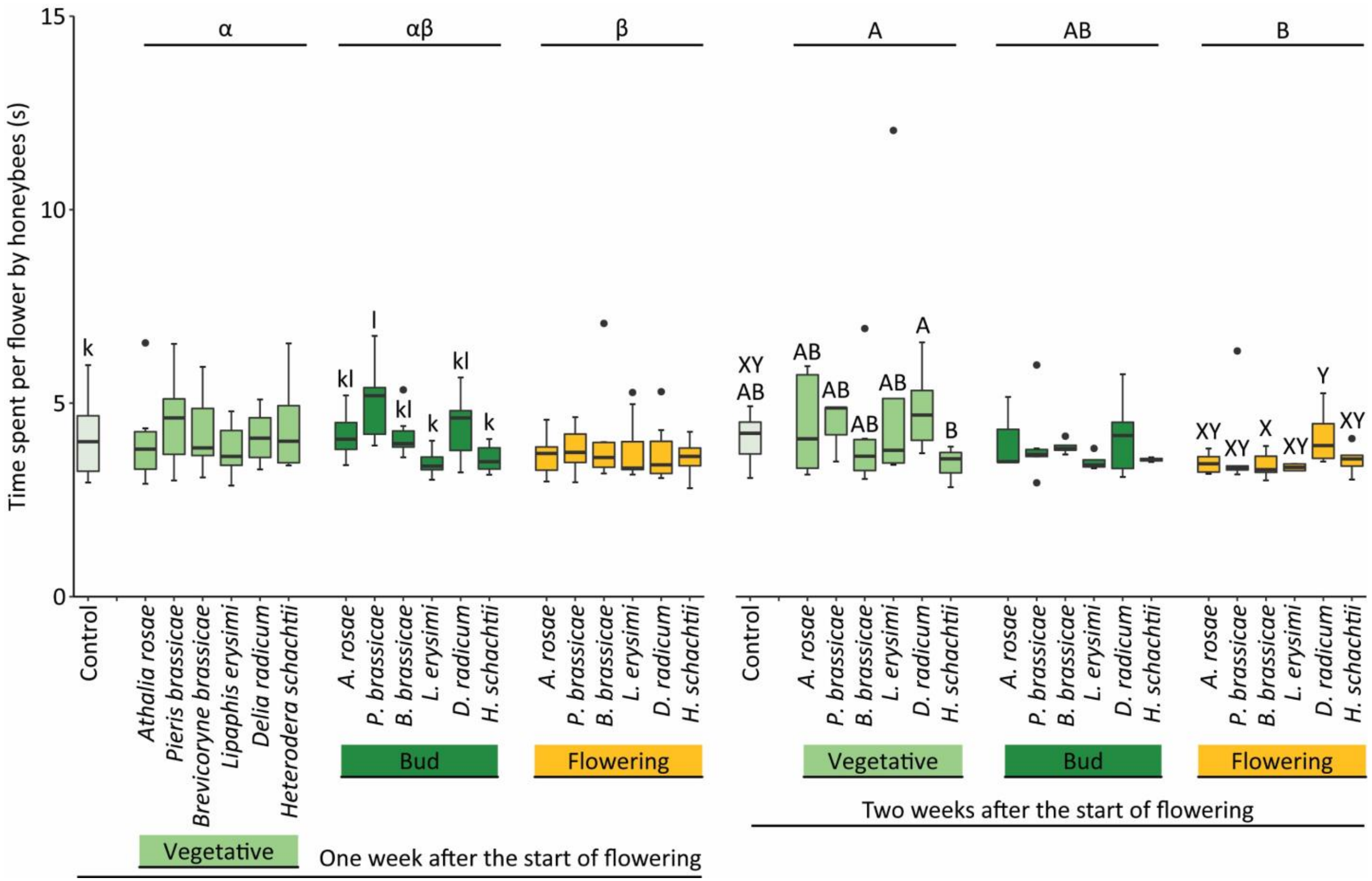

Fig. S13 Time spent per flower by honeybees on flowers of uninfested plots (control) of *Brassica nigra* plants and on flowers of plots infested by herbivores at different plant ontogenetic stages. Boxplots show median (line), 1st and 3rd quartiles, minimum and maximum. Outliers (1.5 times the interquartile range below the 1st or above the 3rd quartile) are represented by circles. Observations lasted for 10 min and were made at two time points: between 7 - 9 days and between 14 - 16 days after plots had started flowering. For 7 - 9 days after plots had started flowering, the number of replicates per herbivore treatment varied between 7 and 9, and was 16 for the control treatment. For 14 - 16 days after plots had started flowering, the number of replicates per herbivore treatment varied between 2 and 6, and was 10 for the control treatment. Letter groups (a - b, k - l, x - y) above bars indicate significant differences at ($P \leq 0.05$) between herbivore species within a plant ontogenetic stage based on Tukey's *post hoc* tests, and small or capital letters were used for different time-points. Greek letters above lines indicate significant differences at ($P \leq 0.05$) between plant ontogenetic stages based on Tukey's *post hoc* tests, whereas *ns* indicates no differences.

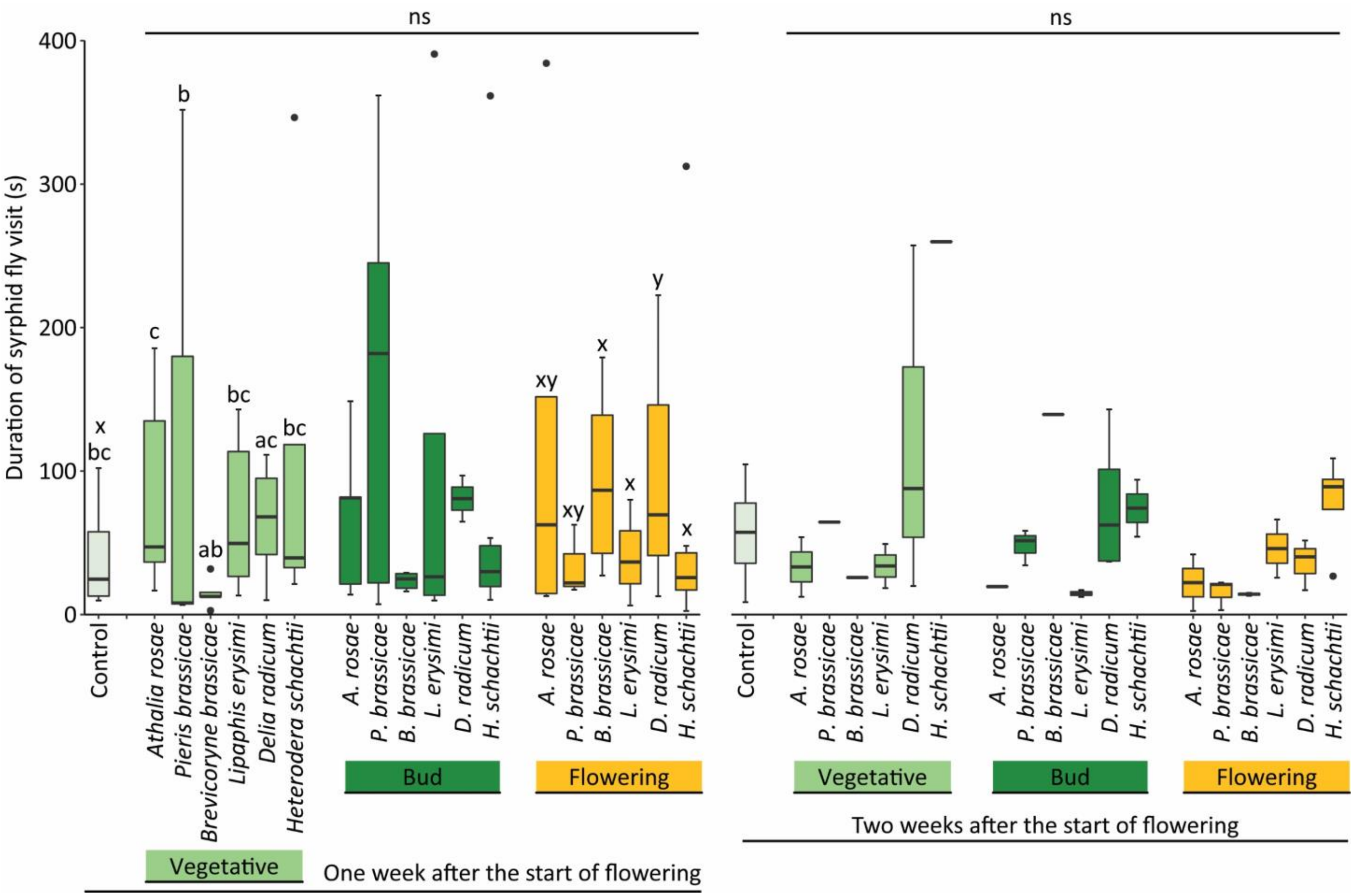

Fig. S14 Duration of syrphid fly visits to uninfested plots (control) of *Brassica nigra* plants and to plots infested by herbivores at different plant ontogenetic stages. Boxplots show median (line), 1st and 3rd quartiles, minimum and maximum. Outliers (1.5 times the interquartile range below the 1st or above the 3rd quartile) are represented by circles. Observations lasted for 10 min and were made at two time points: between 7 – 9 days and between 14 - 16 days after plots had started flowering. For 7 - 9 days after plots had started flowering, the number of replicates per herbivore treatment varied between 2 and 6, and was 8 for the control treatment. For 14 - 16 days after plots had started flowering, the number of replicates per herbivore treatment varied between 0 and 4, and was 6 for the control treatment. Letter groups (a - c, x - y) above bars indicate significant differences at ($P \leq 0.05$) between herbivore species within a plant ontogenetic stage based on Tukey's *post hoc* tests. *ns* indicates no differences between plant ontogenetic stages based on Tukey's *post hoc* tests.

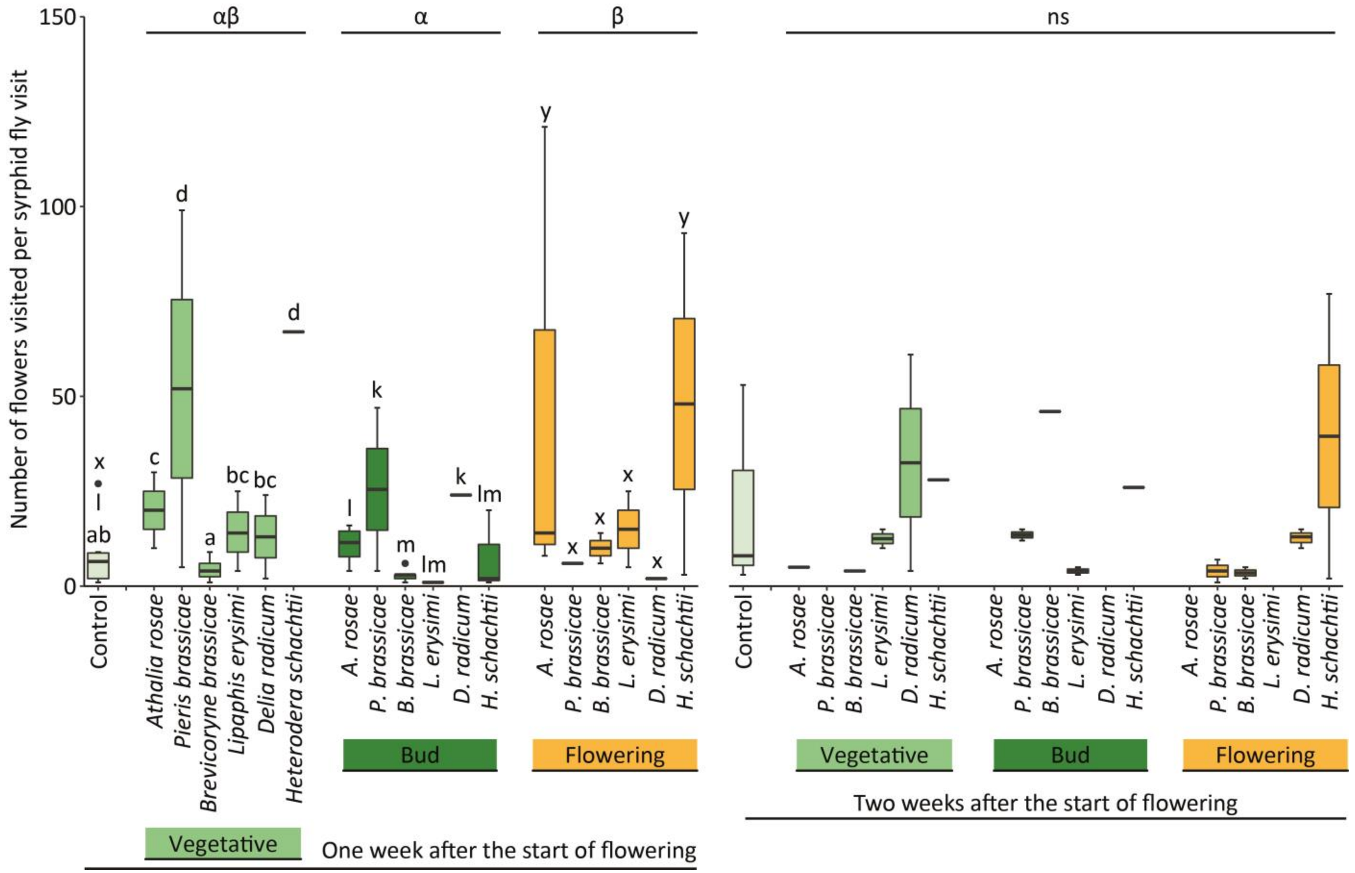

Fig. S15 Number of flowers visited per syrphid fly visit to uninfested plots (control) of *Brassica nigra* plants and to plots infested by herbivores at different plant ontogenetic stages. Boxplots show median (line), 1st and 3rd quartiles, minimum and maximum. Outliers (1.5 times the interquartile range below the 1st or above the 3rd quartile) are represented by circles. Observations lasted for 10 min and were made at two time points: between 7 – 9 days and between 14 - 16 days after plots had started flowering. For 7 - 9 days after plots had started flowering, the number of replicates per herbivore treatment varied between 2 and 6, and was 8 for the control treatment. For 14 - 16 days after plots had started flowering, the number of replicates per herbivore treatment varied between 0 and 4, and was 6 for the control treatment. Letter groups (a - d, k - m, x - y) above bars indicate significant differences at ($P \leq 0.05$) between herbivore species within a plant ontogenetic stage based on Tukey's *post hoc* tests. Greek letters above lines indicate significant differences at ($P \leq 0.05$) between plant ontogenetic stages based on Tukey's *post hoc* tests, whereas *ns* indicates no differences.

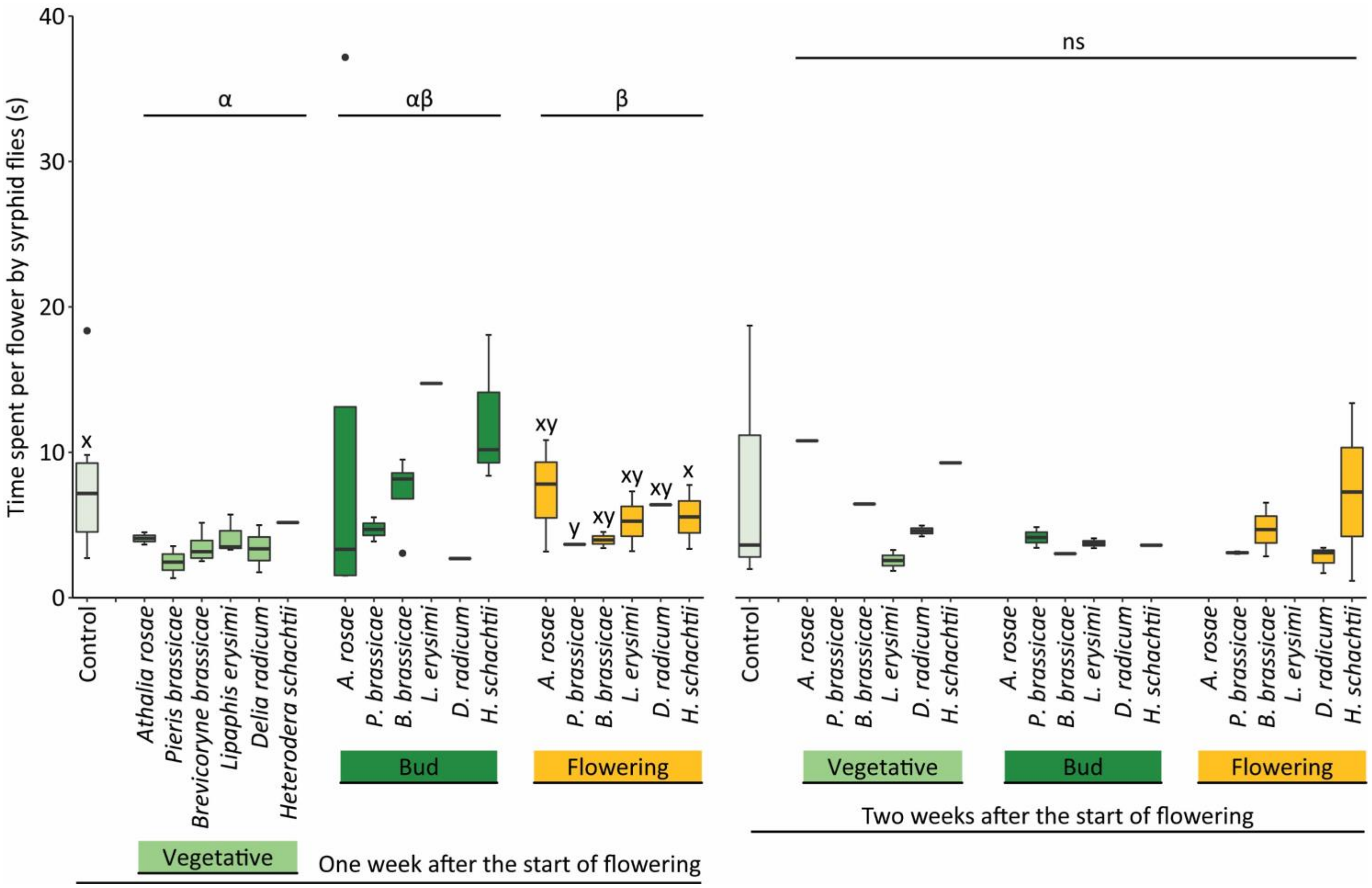

Fig. S16 Time spent per flower by syrphid flies on flowers of uninfested plots (control) of *Brassica nigra* plants and on flowers of plots infested by herbivores at different plant ontogenetic stages. Boxplots show median (line), 1st and 3rd quartiles, minimum and maximum. Outliers (1.5 times the interquartile range below the 1st or above the 3rd quartile) are represented by circles. Observations lasted for 10 min and were made at two time points: between 7 - 9 days and between 14 - 16 days after plots had started flowering. For 7 - 9 days after plots had started flowering, the number of replicates per herbivore treatment varied between 2 and 6, and was 8 for the control treatment. For 14 - 16 days after plots had started flowering, the number of replicates per herbivore treatment varied between 0 and 4, and was 6 for the control treatment. Letter group (x - y) above bars indicate significant differences at ($P \leq 0.05$) between herbivore species within a plant ontogenetic stage based on Tukey's *post hoc* tests. Greek letters above lines indicate significant differences at ($P \leq 0.05$) between plant ontogenetic stages based on Tukey's *post hoc* tests, whereas *ns* indicates no differences.

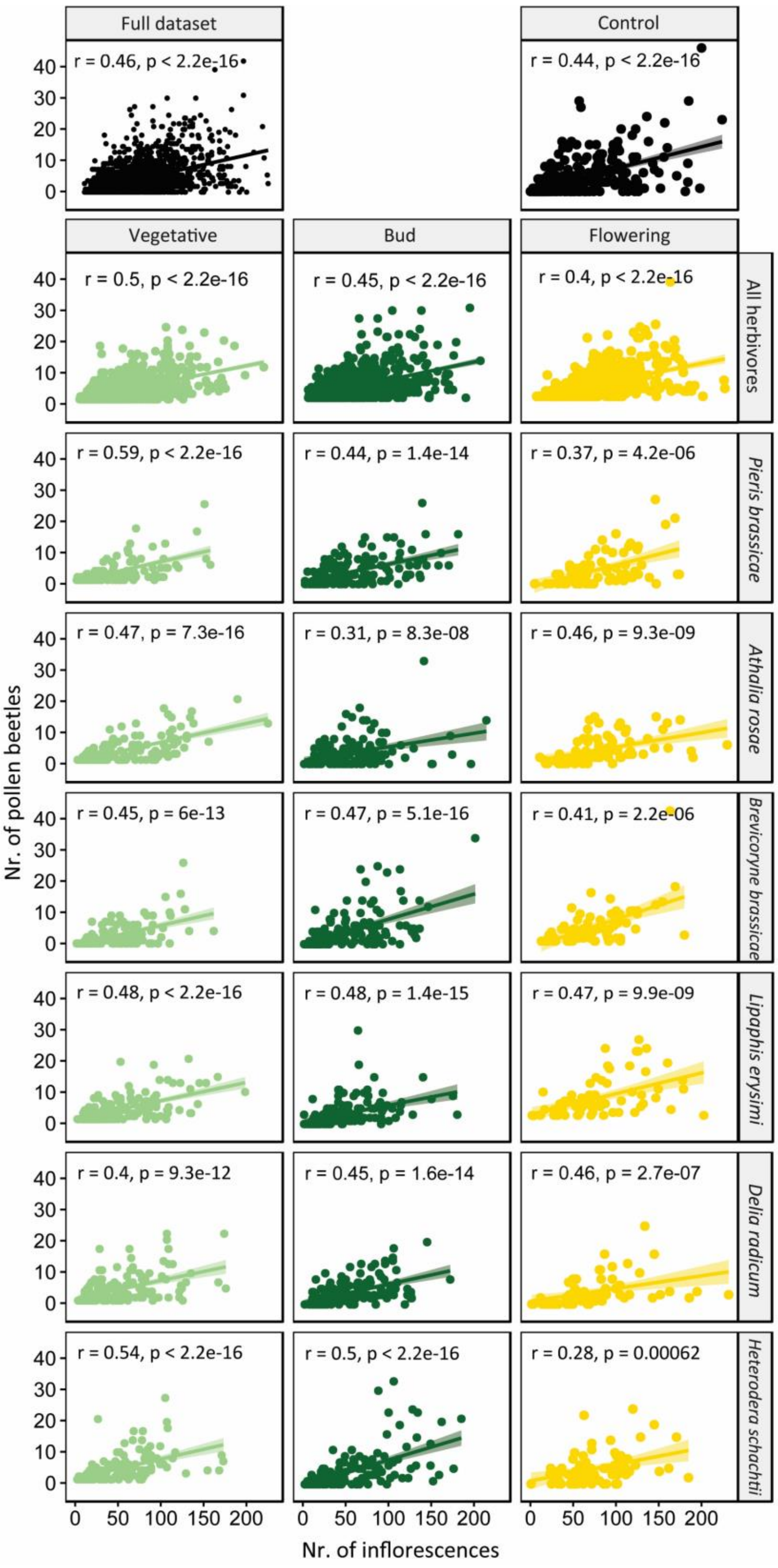

Fig. S17 Number of adult pollen beetles on flowers of *Brassica nigra* plants with different numbers of inflorescences one week after plots had reached the bud stage. Plots were uninfested (control) or infested with herbivores at different plant ontogenetic stages. Correlation coefficient r was computed using the Kendall method.

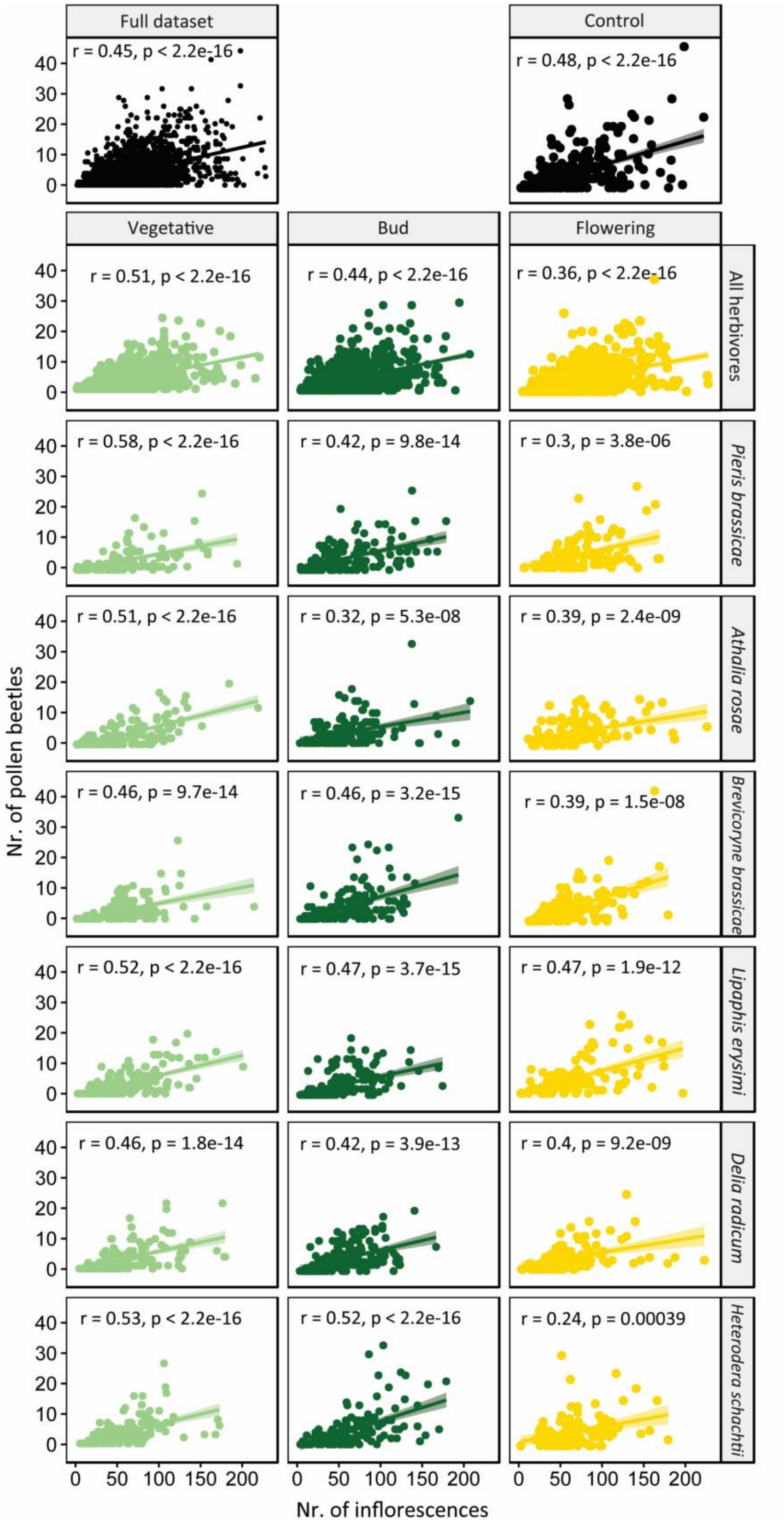

Fig. S18 Number of adult pollen beetles on flowers of *Brassica nigra* plants with different numbers of inflorescences one week after plots had started flowering. Plots were uninfested (control) or infested with herbivores at different plant ontogenetic stages. Correlation coefficient r was computed using the Kendall method.

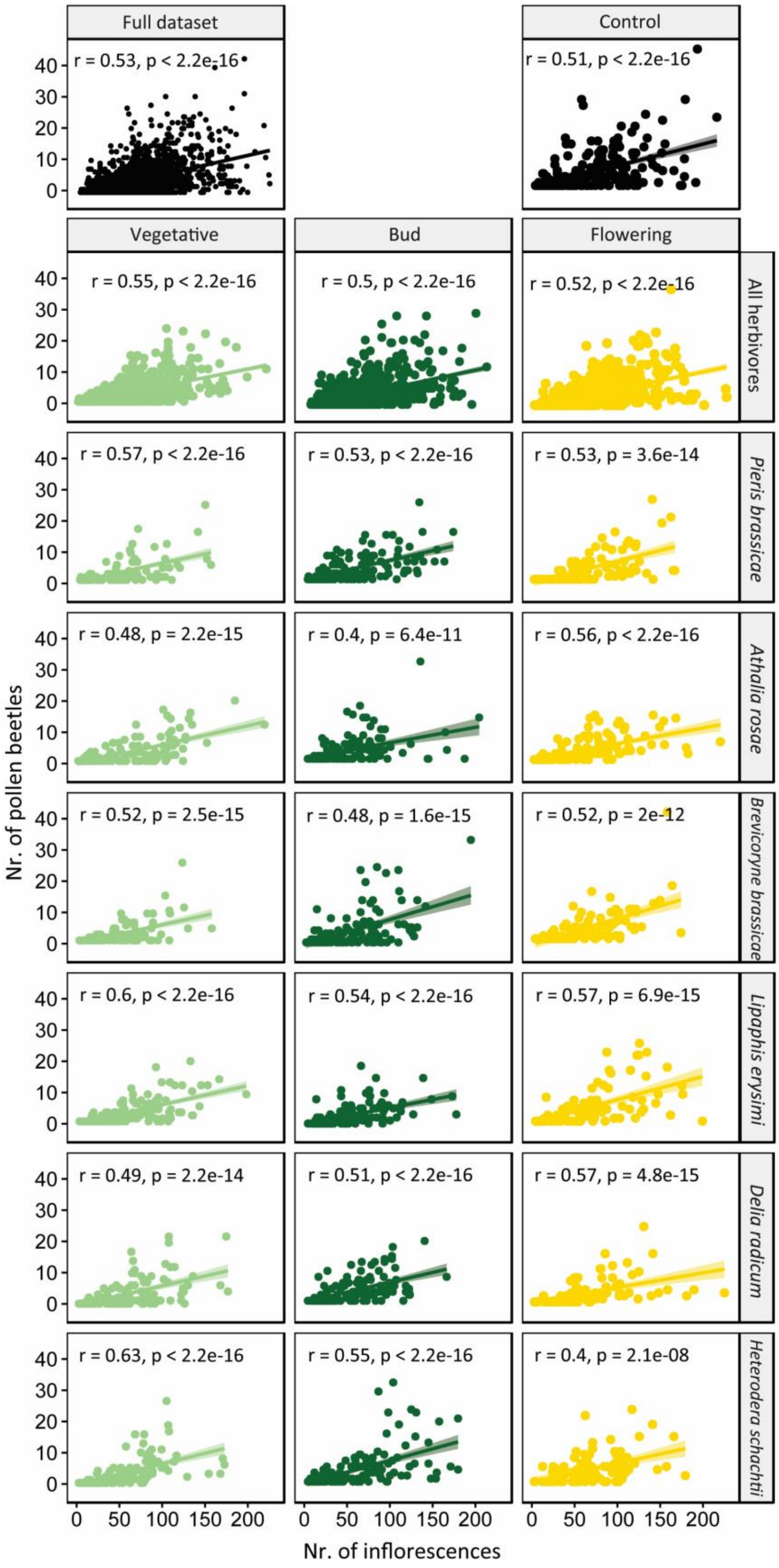

Fig. S19 Number of adult pollen beetles on flowers of *Brassica nigra* plants with different numbers of inflorescences two weeks after plots had started flowering. Plots were uninfested (control) or infested with herbivores at different plant ontogenetic stages. Correlation coefficient r was computed using the Kendall method.

Table S6. Output of generalized linear (mixed) models showing the effects of different fixed (herbivore species, plant ontogenetic stage, and herbivore functional group) factors on plant seed set. All random factors were initially included in the model, and factors which explained less than 3 percent variation or with a *P*-value above 0.05 were excluded from the model. Bold values indicate results where $P \leq 0.05$. Italic values indicate results where $P \leq 0.1$.

	Fixed factors												Random factors					
	Herbivore species (T)			Plant ontogenetic stage (O)			T*O			Herbivore functional group (HFG)			Plot			Plant position		
Number of seeds	df	χ^2	P	df	χ^2	P	df	χ^2	P	df	χ^2	*P*	df	χ^2	P	df	χ^2	P
per plot	5	15.14	**0.010**	2	3.49	0.175	10	28.72	**0.001**	-	-	-	1	7.18	**0.007**	1	26.23	**<0.001**
Vegetative stage	3	19.30	**<0.001**	-	-	-	-	-	-	3	0.47	0.925	1	12.45	**<0.001**	1	24.87	**<0.001**
Bud stage	3	0.06	0.997	-	-	-	-	-	-	3	0.12	0.990	1	0.89	0.347	1	6.96	**0.008**
Flowering stage	3	2.88	0.410	-	-	-	-	-	-	3	2.93	0.403	1	8.42	**0.004**	1	35.46	**<0.001**
per central plant	5	12.70	**0.026**	2	2.94	0.230	10	23.05	**0.011**	-	-	-	-	-	-	-	-	-
Vegetative stage	3	16.78	**<0.001**	-	-	-	-	-	-	3	6.97	*0.073*	-	-	-	-	-	-
Bud stage	3	3.51	0.320	-	-	-	-	-	-	3	3.42	0.331	-	-	-	-	-	-
Flowering stage	3	0.82	0.844	-	-	-	-	-	-	3	1.94	0.584	-	-	-	-	-	-
per side plant	5	11.83	**0.037**	2	8.51	**0.014**	10	19.39	**0.036**	-	-	-	1	4.08	**0.043**	-	-	-
Vegetative stage	3	19.76	**<0.001**	-	-	-	-	-	-	3	4.78	0.189	1	1.23	0.268	-	-	-
Bud stage	3	0.49	0.921	-	-	-	-	-	-	3	0.35	0.950	1	0	1	-	-	-
Flowering stage	3	8.56	**0.036**	-	-	-	-	-	-	3	3.28	0.351	1	1.45	0.229	-	-	-
Weight of 100 seeds	5	26.54	**<0.001**	2	9.14	**0.010**	10	29.78	**<0.001**	-	-	-	-	-	-	-	-	-
Vegetative stage	3	24.07	**<0.001**	-	-	-	-	-	-	3	14.13	**0.003**	-	-	-	-	-	-
Bud stage	3	2.60	0.458	-	-	-	-	-	-	3	6.01	0.111	-	-	-	-	-	-
Flowering stage	3	3.30	0.348	-	-	-	-	-	-	3	7.41	*0.060*	-	-	-	-	-	-

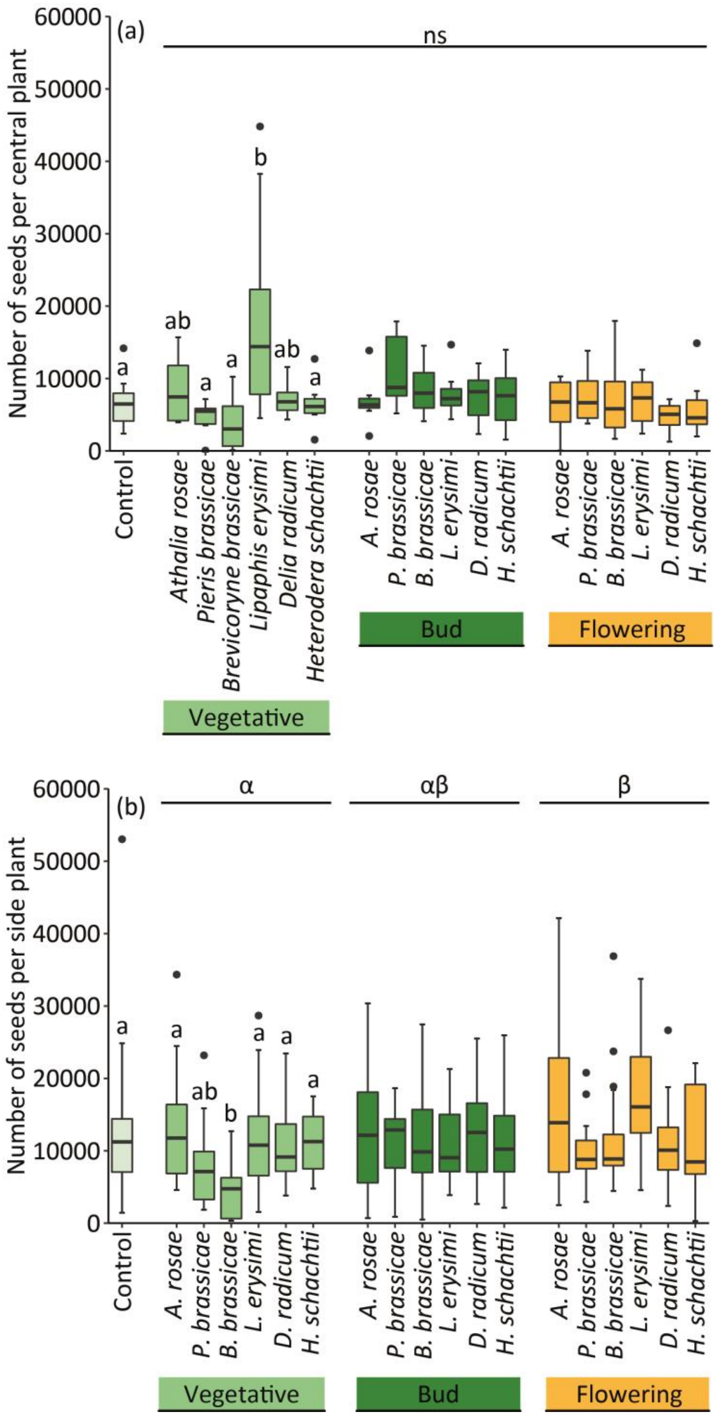

Fig. S20 Number of seeds produced by uninfested (control) *Brassica nigra* plants and plants infested by herbivores at different plant ontogenetic stages. We assessed the number of seeds produced per central plant (a) and per side plant (b). Boxplots show median (line), 1st and 3rd quartiles, minimum and maximum. Outliers (1.5 times the interquartile range below the 1st or above the 3rd quartile) are represented by circles. For central plants, the number of replicates per herbivore treatment varied between 14 and 17, and was 28 for uninfested plants. For side plants, the number of replicates per herbivore treatment varied between 29 and 35, and was 63 for uninfested plants. Letters above bars indicate significant differences at ($P \leq 0.05$) between herbivore species within a plant ontogenetic stage based on Tukey's *post hoc* tests. Greek letters above lines indicate significant differences at ($P \leq 0.05$) between plant ontogenetic stages based on Tukey's *post hoc* tests, whereas *ns* indicates no differences.
